# Concerted regulation of non-alcoholic fatty liver disease progression by microRNAs in apolipoprotein E-deficient mice

**DOI:** 10.1242/dmm.049173

**Published:** 2021-12-24

**Authors:** Andrea R. López-Pastor, Jorge Infante-Menéndez, Tamara González-Illanes, Paula González-López, Águeda González-Rodríguez, Carmelo García-Monzón, Melina Vega de Céniga, Leticia Esparza, Almudena Gómez-Hernández, Óscar Escribano

**Affiliations:** 1 Laboratory of Hepatic and Cardiovascular Diseases, Biochemistry and Molecular Biology Department, School of Pharmacy, Complutense University of Madrid, 28040 Madrid, Spain; 2 Liver Research Unit, Hospital Universitario Santa Cristina, Instituto de Investigación Sanitaria Princesa, 28009 Madrid, Spain; 3 CIBER of Hepatic and Digestive Diseases (CIBERehd), 28029 Madrid, Spain; 4 Department of Angiology and Vascular Surgery, Hospital de Galdakao-Usansolo, Galdakao, 48960 Bizkaia, Spain; 5 Biocruces Bizkaia Health Research Institute, Barakaldo, 48903 Bizkaia, Spain

**Keywords:** Non-alcoholic fatty liver disease, MicroRNAs, Lipid metabolism, Insulin resistance, Autophagy, Extracellular vesicles

## Abstract

The prevalence of non-alcoholic fatty liver disease (NAFLD) is constantly increasing, and altered expression of microRNAs (miRNAs) fosters the development and progression of many pathologies, including NAFLD. Therefore, we explored the role of new miRNAs involved in the molecular mechanisms that trigger NAFLD progression and evaluated them as biomarkers for diagnosis. As a NAFLD model, we used apolipoprotein E-deficient mice administered a high-fat diet for 8 or 18 weeks. We demonstrated that insulin resistance and decreased lipogenesis and autophagy observed after 18 weeks on the diet are related to a concerted regulation carried out by miR-26b-5p, miR-34a-5p, miR-149-5p and miR-375-3p. We also propose circulating let-7d-5p and miR-146b-5p as potential biomarkers of early stages of NAFLD. Finally, we confirmed that circulating miR-34a-5p and miR-375-3p are elevated in the late stages of NAFLD and that miR-27b-3p and miR-122-5p are increased with disease progression. Our results reveal a synergistic regulation of key processes in NAFLD development and progression by miRNAs. Further investigation is needed to unravel the roles of these miRNAs for developing new strategies for NAFLD treatment.

This article has an associated First Person interview with the joint first authors of the paper.

## Abstract (precis)

**Summary:** *Apoe^−/−^* mice administered a high-fat diet represent a model of non-alcoholic fatty liver disease, revealing the synergistic regulation of key processes in disease progression by miRNAs and indicating some miRNAs as biomarkers for diagnosis.

## INTRODUCTION

Non-alcoholic fatty liver disease (NAFLD) is considered the most common hepatic disorder in Western countries, where its prevalence is continuously growing (Wree et al., 2013; Wong et al., 2014; McPherson et al., 2015; Rinella and Sanyal, 2016; Younossi et al., 2018). This condition represents the hepatic hallmark of metabolic syndrome, because it is strongly related to obesity, insulin resistance, dyslipidemia and hypertension (Younossi et al., 2018). Hence, owing to the research of recent years and the heterogeneity, experts have considered that the term NAFLD is not accurate enough, and the name metabolic dysfunction-associated fatty liver disease (MAFLD) has been proposed (Eslam et al., 2020; Tilg and Effenberger, 2020). NAFLD is defined by the accumulation of fat in the liver, and it consists of two main stages, the earliest one characterized by benign steatosis in >5% of hepatocytes (fatty liver) that can progress to non-alcoholic steatohepatitis (NASH) [European Association for the Study of the Liver (EASL), European Association for the Study of Diabetes (EASD) and European Association for the Study of Obesity (EASO), 2016; Arab et al., 2018; Friedman et al., 2018]. The hallmark of this severe stage is hepatocyte ballooning, inflammation and/or fibrosis, eventually resulting in hepatic cirrhosis and, ultimately, hepatocellular carcinoma [Marra et al., 2008; European Association for the Study of the Liver (EASL), European Association for the Study of Diabetes (EASD) and European Association for the Study of Obesity (EASO), 2016; Friedman et al., 2018; Pierantonelli and Svegliati-Baroni, 2019]. Owing to the high prevalence mentioned above, although hepatitis C is the leading cause of liver transplantation, it is expected that NASH will exceed it in the near future (Wree et al., 2013; Wong et al., 2014; Rinella and Sanyal, 2016). Although some factors involved in the progression from steatosis to NASH are still unknown, the roles of lipotoxicity, oxidative stress and activation of the immune system have been well characterized (Vernon et al., 2011). Considering these data, it is essential to gain deeper knowledge about the molecular mechanisms involved in NAFLD, with the aim of discovering new targets for treatment.

In recent years, microRNAs (miRNAs) have been proposed as novel and interesting tools for this purpose, belonging to an evolutionarily conserved class of short (20-22 nucleotides in length) and single-stranded non-coding RNAs. In mammals, the majority of gene expression is repressed at the post-transcriptional level by miRNAs (Hussain, 2012; Hartig et al., 2015). Particularly, owing to the complementarity between these molecules and their targets, miRNAs are able to associate with the 3′ untranslated region (UTR) of a gene mRNA, leading to mRNA decay and/or translational repression (Huntzinger and Izaurralde, 2011). Consequently, they have a critical role in a huge number of physiological processes, such as cell growth, tissue differentiation and embryonic development. Indeed, their alteration is involved in the onset and progression of pathological states, including obesity, NAFLD and cardiovascular diseases (Hussain, 2012; Hartig et al., 2015). Currently, the major drawback of miRNAs is poor understanding of the mechanisms by which they exert their actions. A concerted effort is needed before manipulation of miRNA expression can be used in disease management worldwide.

Aberrant miRNA expression in both liver and plasma has been described as a main feature of liver diseases in many studies, including NAFLD (Yokota et al., 2001; Madan et al., 2006; Bettaieb et al., 2015). In this sense, extracellular vesicles (EVs), mainly exosomes, are able to vehiculize miRNAs mediating tissue crosstalk while protecting them from degradation by RNAses. Therefore, circulating miRNAs are being considered as potential biomarkers in the diagnosis and prognosis of many pathologies, such as NAFLD, cancer, cardiovascular diseases, obesity and diabetes (Mori et al., 2019).

With this background, we used a mouse deficient in apolipoprotein E (*Apoe*) and administered a high-fat diet (HFD) as a model of NAFLD. The principal aim of the present work was to determine whether dysregulation of the expression of certain miRNAs could trigger NAFLD development. With this objective in mind, we tried to identify molecular targets of these miRNAs as key proteins involved in fostering disease progression. Finally, we also analyzed EVs in order to find miRNAs that could serve as NAFLD biomarkers.

## RESULTS

### Evaluation of weight gain and lipid profile

**Fig. 1.**
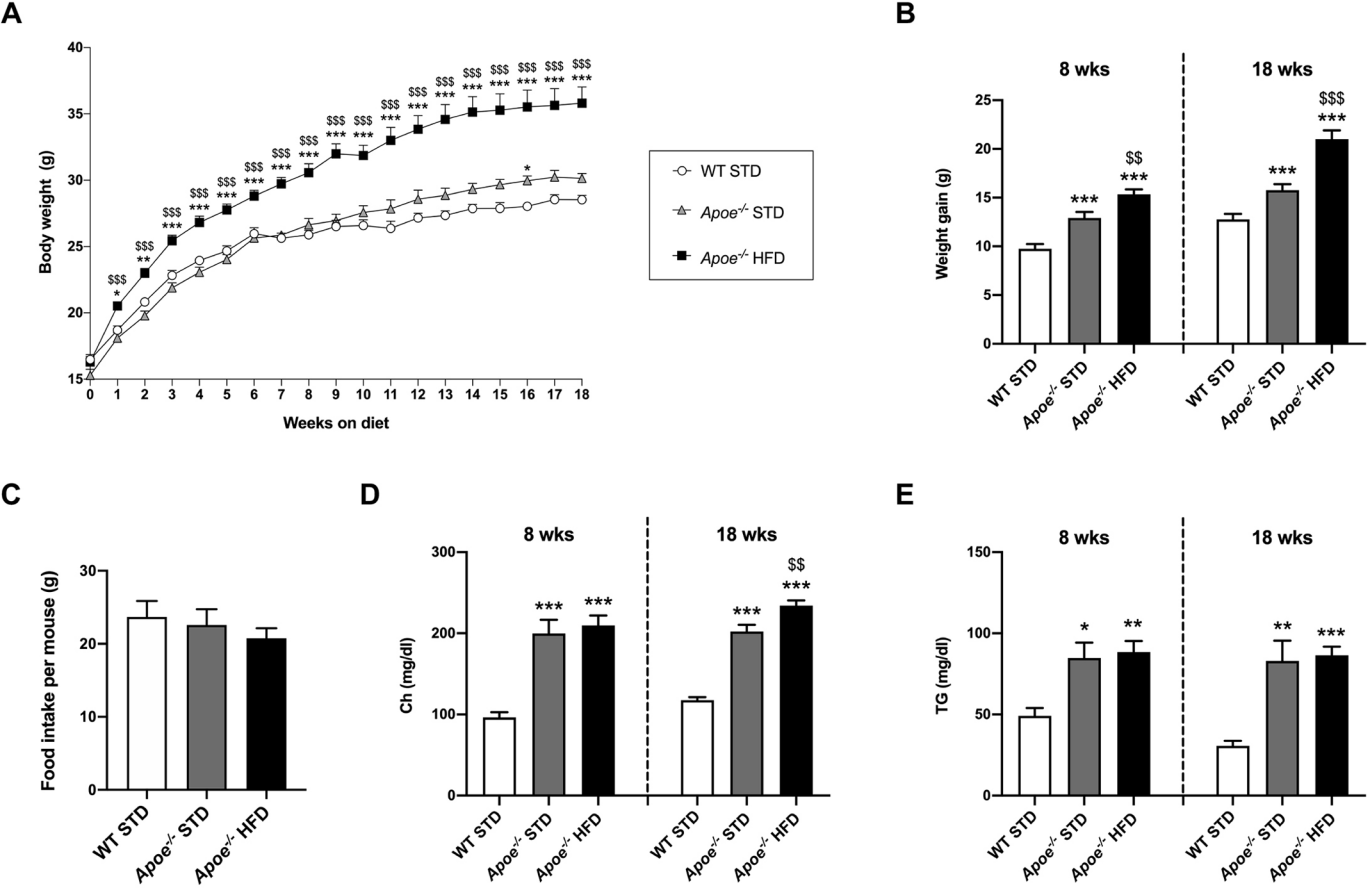
**Characterization of obesity and lipid profile in the mouse model of NAFLD.** (A) Graph showing BW progression in STD-fed WT (WT STD; *n*=20-39), STD-fed *Apoe^−/−^* (*Apoe^−/−^* STD; *n*=10-37) and HFD-fed *Apoe^−/−^* (*Apoe^−/−^* HFD; *n*=18-38) mice from weaning until the 18th week on the diet. (B) Representation of BW gain from weaning to 8 or 18 weeks of diet in the three experimental groups. (C) Assessment of food intake per mouse and per week (g) in WT STD (*n*=16), *Apoe^−/−^* STD (*n*=23) and *Apoe^−/−^* HFD (*n*=27) mice. (D) Levels of plasma Ch (mg/dl) in WT STD (*n*=6-7), *Apoe^−/−^* STD (*n*=5-9) and *Apoe^−/−^* HFD (*n*=8-10) mice after 8 or 18 weeks on the diet. (E) Circulating TG levels (mg/dl) in WT STD (*n*=7), *Apoe^−/−^* STD (*n*=4-9) and *Apoe^−/−^* HFD (*n*=8-10) mice after 8 or 18 weeks on the diet. Results are expressed as mean±s.e.m. Statistical significance was assessed by two-tailed unpaired Student's *t*-test, except for TGs at 8 weeks of diet, which were evaluated by unpaired non-parametric Mann–Whitney U test. *P<0.05, **P<0.01 and ***P<0.001 versus WT STD mice; ^$$^*P*<0.01 and ^$$$^*P*<0.001 versus *Apoe^−/−^* STD mice. BW, body weight; Ch, cholesterol; HFD, high-fat diet; STD, standard diet; TG, triglycerides; WT, wild type.

After genotype characterization, wild-type (WT) and apolipoprotein E-deficient (*Apoe^−/−^*) mice were administered a standard diet (STD) or HFD for 8 or 18 weeks (Fig. S1). In order to study the diet-associated weight gain, we evaluated body weight (BW) and gain from weaning until euthanasia (Fig. 1A,B), BW at the time of euthanasia, and weights of liver, subcutaneous fat, represented by inguinal white adipose tissue (iWAT) and visceral fat (vWAT), normalized versus BW (Table 1). According to the results, BW, weight gain and adipose depots were significantly increased, while the liver/BW ratio was significantly diminished, in the HFD-fed *Apoe^−/−^* versus STD-fed *Apoe^−/−^* and WT groups (Fig. 1A,B and Table 1). Additionally, food intake was measured; however, no statistically significant differences were observed when comparing the three groups (Fig. 1C).

**Table 1.**
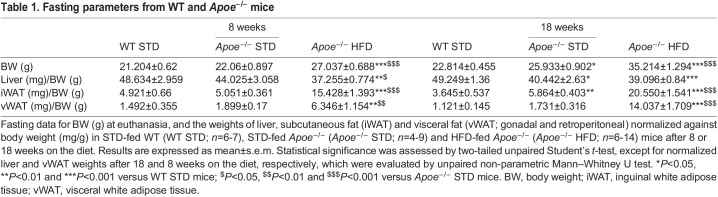
Fasting parameters from WT and *Apoe^−/−^* mice

To obtain the plasma lipid profile, cholesterol (Ch) and triglyceride (TG) levels were measured. The circulating levels of Ch and TGs were significantly higher in *Apoe^−/−^* mice than in the WT groups (Fig. 1D,E). Furthermore, 18-week HFD-fed *Apoe^−/−^* mice had significantly higher plasma Ch levels than STD-fed *Apoe^−/−^* mice (Fig. 1D).

**Fig. 2.**
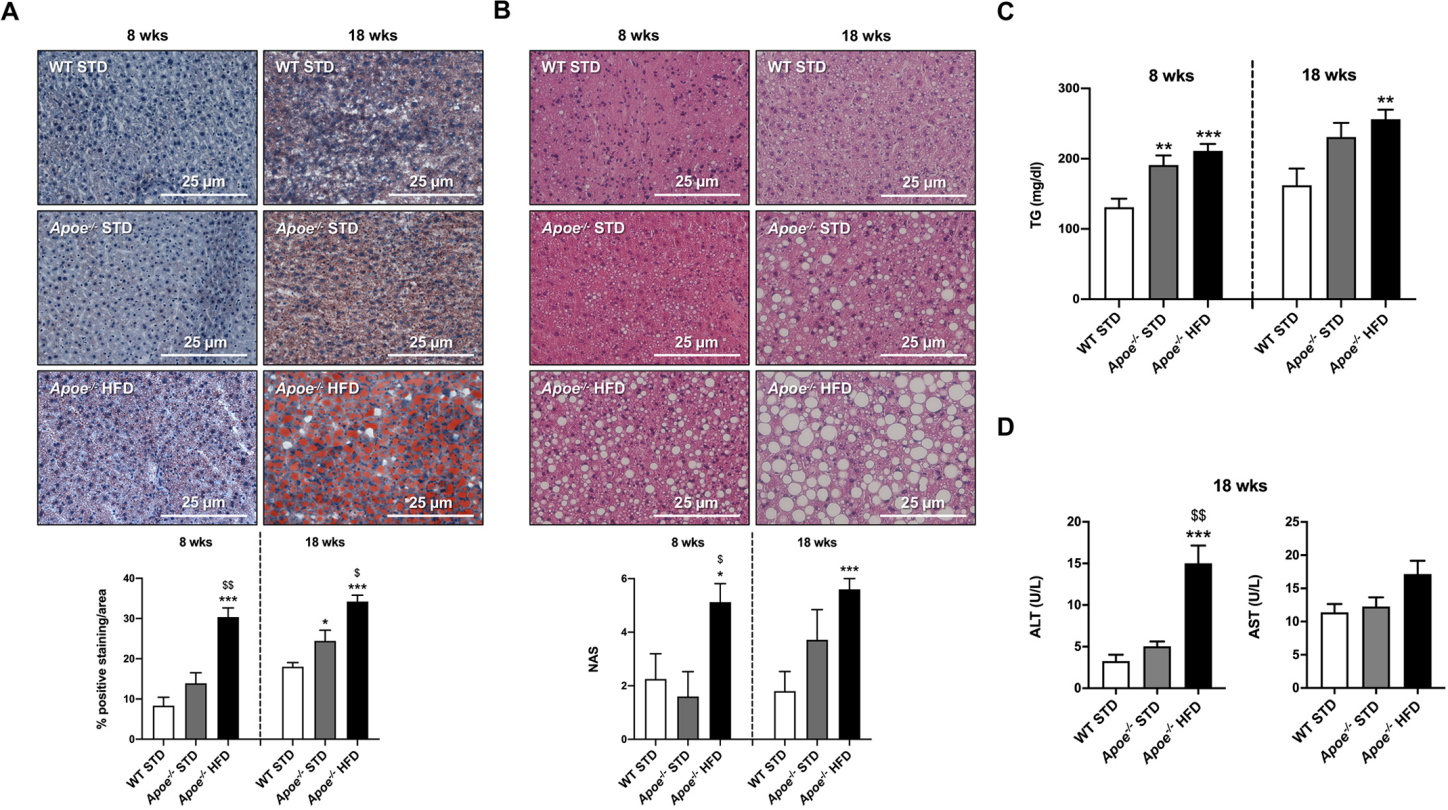
**Assessment of intrahepatic lipid accumulation and NAS.** (A) Representative images of Oil Red O staining to evaluate hepatic-specific lipid content (top; image magnification ×20), and its quantification in WT STD (*n*=6-7), *Apoe^−/−^* STD (*n*=5-6) and *Apoe^−/−^* HFD (*n*=5-6) mice after 8 or 18 weeks on the diet (bottom). (B) Representative images of H&E staining to perform liver histological analysis (top; image magnification ×20), and quantification of NAS according to liver abnormalities in WT STD (*n*=4-5), *Apoe^−/−^* STD (*n*=5-7) and *Apoe^−/−^* HFD (*n*=8-10) mice after 8 or 18 weeks on the diet (bottom). (C) Intrahepatic TG quantification (mg/dl) in WT STD (*n*=6-7), *Apoe^−/−^* STD (*n*=5-6) and *Apoe^−/−^* HFD (*n*=6-8) mice after 8 or 18 weeks on the diet. (D) Quantification of both types of circulating transaminases (ALT, left; AST, right; U/L) in WT STD (*n*=6-7), *Apoe^−/−^* STD (*n*=7-8) and *Apoe^−/−^* HFD (*n*=9) mice after 18 weeks on the diet. Results are expressed as mean±s.e.m. Statistical significance was assessed by two-tailed unpaired Student's *t*-test, except for plasma ALT measurement, which was evaluated by unpaired non-parametric Mann–Whitney U test. *P<0.05, **P<0.01 and ***P<0.001 versus WT STD mice; ^$^*P*<0.05 and ^$$^*P*<0.01 versus *Apoe^−/−^* STD mice. ALT, alanine transaminase; AST, aspartate transaminase; NAS, NAFLD Activity Score; TG, triglycerides.

Given that *Apoe* deficiency induces NAFLD, in order to investigate the liver tissue and possible lipid ectopic depot, we evaluated hepatic lipid accumulation by Oil Red O staining in liver sections from *Apoe^−/−^* and WT mice. The results revealed a significant increase in the percentage Oil Red O-positive area in HFD-fed mice compared with STD-fed mice after 8 and 18 weeks on the respective diets. Moreover, in the case of 18-week STD-fed *Apoe^−/−^* mice, a statistically significant increase was observed with respect to the STD-fed WT group (Fig. 2A).

In a further step, we also checked the hepatic morphological changes through Hematoxylin and Eosin (H&E) staining in other sections. The results were evaluated by a pathologist specializing in hepatic histopathology from Santa Cristina Hospital (Madrid, Spain), using the NAFLD Activity Score (NAS) scoring system as a tool and following the established criteria (Kleiner et al., 2005). These data suggested that 8-week HFD-fed *Apoe^−/−^* mice presented a higher NAS with respect to that of the STD-fed groups. However, this increase was only statistically significant versus STD-fed WT mice after 18 weeks on the diet (Fig. 2B). The breakdown of these parameters in steatosis, inflammation and ballooning, together with the mRNA levels of the chemokine monocyte chemoattractant protein-1 (*Mcp1*), also known as *Ccl2*, are shown in Fig. S2. Regarding *Ccl2* gene expression, levels were significantly higher in the HFD-fed *Apoe^−/−^* mice than in the STD-fed *Apoe^−/−^* and WT mice (Fig. S2B).

In addition, lipid droplet size was measured, revealing that the lipid droplet size of 8-week HFD-fed *Apoe^−/−^* mice was significantly larger than that of STD-fed WT mice. Furthermore, lipid droplet size was markedly increased in HFD-fed *Apoe^−/−^* mice after 18 weeks on the diet, compared with that in both groups of STD-fed mice, and this difference was statistically significant. Moreover, the lipid droplet size in STD-fed *Apoe^−/−^* mice was significantly larger than that in STD-fed WT mice after 18 weeks on the diet (Fig. S2C).

NASH is characterized not only by the presence of hepatic steatosis and inflammation, but also fibrosis. Thus, we performed Sirius Red staining in liver sections of 18-week-fed mice to determine hepatic fibrosis. Quantification of positive staining revealed the highest levels of fibrosis in 18-week HFD-fed *Apoe^−/−^* mice, which were significantly different from those in STD-fed mice (Fig. S2D). The results were also evaluated by the pathologist mentioned above, using the established criteria of fibrosis scoring (Liang et al., 2014). These data suggested that 18-week HFD-fed *Apoe^−/−^* mice presented a statistically higher fibrosis score than that of STD-fed WT mice. However, our findings only indicated an initial stage of hepatic fibrosis (Fig. S2D).

Intrahepatic TG concentrations were also measured in our experimental groups after 8 and 18 weeks on the respective diets. Our results showed significantly higher intrahepatic TG concentration in 8-week HFD- and STD-fed *Apoe^−/−^* mice than in STD-fed WT mice, but, after 18 weeks on the respective diets, this difference was only statistically significant between the HFD-fed *Apoe^−/−^* and STD-fed WT groups (Fig. 2C).

Next, we assessed the extent of hepatocellular injury through the determination of plasma activities of alanine and aspartate transaminases (ALT and AST, respectively) after 18 weeks of diet administration. Regarding ALT, the most specific marker of liver damage, a statistically significant increase was observed in HFD-fed *Apoe^−/−^* mice compared with the STD-fed *Apoe^−/−^* and WT mice. Concerning plasma AST levels, there were no statistically significant differences among the groups (Fig. 2D).

### Lipogenesis impairment as NAFLD progresses

**Fig. 3.**
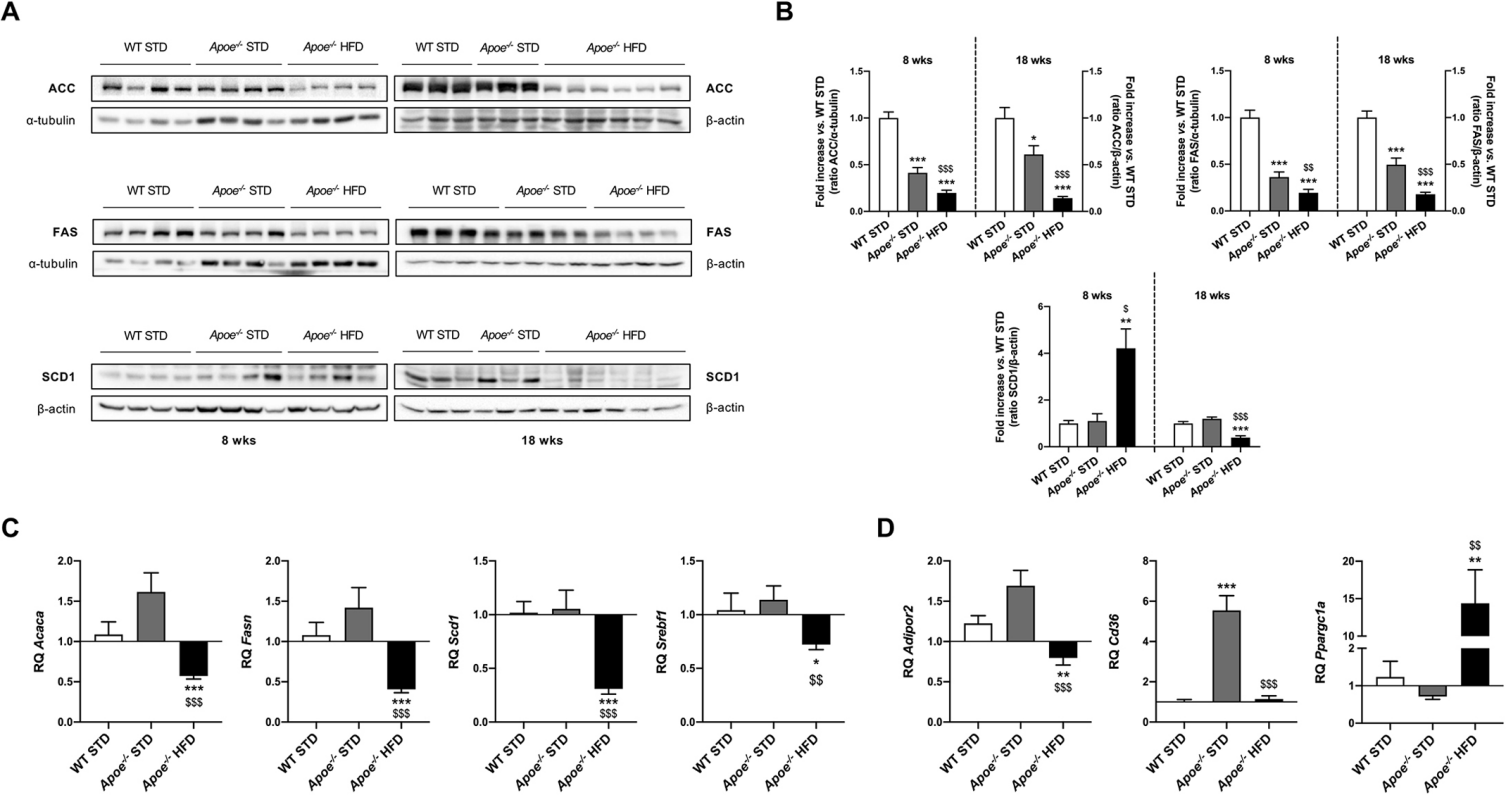
**Expression of pivotal proteins and mRNA levels of genes involved in hepatic lipid metabolism.** (A) Representative western blot analysis of ACC, FAS and SCD1 in liver homogenates from the six groups studied. α-tubulin or β-actin was used as a loading control. (B) Histograms presenting the protein/α-tubulin or β-actin ratio quantifications of band intensities in WT STD (*n*=3-4), *Apoe^−/−^* STD (*n*=3-4) and *Apoe^−/−^* HFD (*n*=4-6) mice after 8 or 18 weeks on the diet. (C) mRNA levels of *Acaca*, *Fasn*, *Scd1* and *Srebf1* by RT-qPCR in livers from WT STD (*n*=5-7), *Apoe^−/−^* STD (*n*=5-8) and *Apoe^−/−^* HFD (*n*=9-14) mice after 18 weeks on the diet. *Actb* was used as a control. (D) mRNA levels of *Adipor2*, *Cd36* and *Ppargc1a* by RT-qPCR in livers from WT STD (*n*=4-5), *Apoe^−/−^* STD (*n*=4-5) and *Apoe^−/−^* HFD (*n*=7-9) mice after 18 weeks on the diet. *Actb* was used as a control. Results are expressed as mean±s.e.m. Statistical significance was assessed by two-tailed unpaired Student's *t*-test, apart from ACC, 8-week-diet FAS and 18-week-diet SCD1 data, and mRNA levels of *Cd36* and *Ppargc1a*, which were evaluated by unpaired non-parametric Mann–Whitney U test. *P<0.05, **P<0.01 and ***P<0.001 versus WT STD mice; ^$^*P*<0.05, ^$$^*P*<0.01 and ^$$$^*P*<0.001 versus *Apoe^−/−^* STD mice.

As has been previously verified, hepatic lipid metabolism dysregulation prevails in NAFLD (Arab et al., 2018; Friedman et al., 2018). Expression of key proteins involved in lipid metabolism and NAFLD progression, such as acetyl-CoA carboxylase (ACC; encoded by *Acaca*), fatty acid synthase (FAS; encoded by *Fasn*) and stearoyl-CoA desaturase 1 (SCD1), were analyzed by western blotting using α-tubulin or β-actin as normalizers (Fig. 3A,B). Hepatic ACC and FAS expression presented the same profile after 8 and 18 weeks of diet, reducing their levels as NAFLD progressed. The levels of these proteins were significantly reduced in both *Apoe^−/−^* groups compared with the WT group, and statistically significant differences were also observed between the two *Apoe^−/−^* groups (Fig. 3A,B). Regarding SCD1 expression, statistically significant changes were only observed in the HFD-fed *Apoe^−/−^* group, in which expression was significantly higher than in the STD-fed groups after 8 weeks on the diet but significantly lower after 18 weeks on the diet (Fig. 3A,B). Significant decrease in the expression of *Acaca*, *Fasn* and *Scd1* in *Apoe^−/−^* mice after 18 weeks of HFD was also demonstrated by reverse transcription and quantitative PCR (RT-qPCR). In addition, we analyzed the mRNA levels of the upstream regulator of lipogenic gene expression, sterol-regulatory element binding protein-1c (SREBP1c; encoded by *Srebf1*), after 18 weeks of diet, and we found a statistically significant downregulation in the HFD-fed *Apoe^−/−^* group compared with the STD-fed *Apoe^−/−^* and WT groups (Fig. 3C).

Furthermore, mRNA levels of other key genes were analyzed by RT-qPCR in the liver of 18-week-fed mice in order to elucidate the mechanisms involved in the onset of NAFLD. In the case of adiponectin receptor 2 (*Adipor2*) mRNA, we also observed significantly reduced expression in HFD-fed *Apoe^−/−^* mice compared with STD-fed *Apoe*^−/−^ and WT mice. However, significantly higher levels of cluster of differentiation 36 (*Cd36*) mRNA expression were observed in STD-fed *Apoe*^−/−^ mice compared with STD-fed WT mice and HFD-fed *Apoe*^−/−^ mice, which presented similar levels. Peroxisome proliferator-activated receptor gamma coactivator 1-alpha (*Ppargc1a*) mRNA expression was significantly higher in the HFD-fed *Apoe*^−/−^ mice than in STD-fed *Apoe*^−/−^ and WT mice (Fig. 3D).

### Assessment of diet-induced insulin resistance

**Fig. 4.**
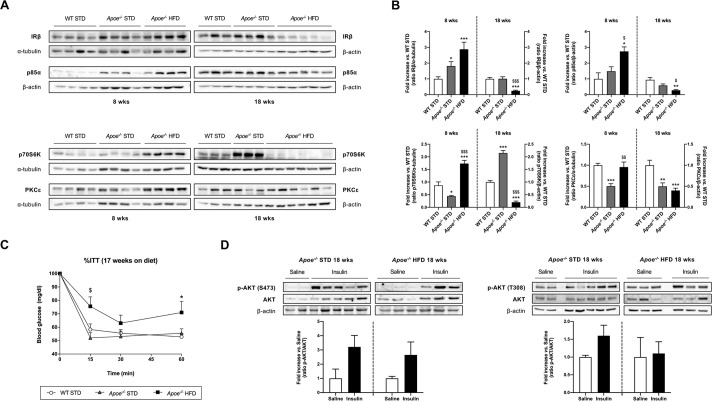
**Evaluation of alterations in insulin signaling.** (A) Representative gels of western blot analysis of IRβ, p85α, p70S6K and PKCε in liver homogenates from the six groups studied (*n*=3-6 per group). α-tubulin or β-actin was used as a loading control. (B) Histogram displaying the protein/α-tubulin or β-actin ratio quantification of band intensities in WT STD (*n*=3-4), *Apoe^−/−^* STD (*n*=3-4) and *Apoe^−/−^* HFD (*n*=4-6) mice after 8 or 18 weeks on the diet. (C) Insulin tolerance test (ITT) in WT STD (*n*=7), *Apoe^−/−^* STD (*n*=6) and *Apoe^−/−^* HFD (*n*=7) animals after 17 weeks on the diet. (D) Western blot analysis of total AKT and the phosphorylated forms (Ser473, left; Thr308, right) in the liver of mice subjected to *in vivo* insulin signaling analysis after 18 weeks on the diet. Top: representative gels of *Apoe^−/−^* STD and *Apoe^−/−^* HFD mice injected with saline (*n*=2-3) or insulin (*n*=3-5) before euthanasia. In both cases, β-actin was used as a loading control. Bottom: histogram showing the normalized phospho-AKT (p-AKT)/AKT ratio in *Apoe^−/−^* STD (*n*=2-3) and *Apoe^−/−^* HFD (*n*=3-5) mice. Results are expressed as mean±s.e.m. Statistical significance was assessed by two-tailed unpaired Student's *t*-test, apart from IRβ and 8-week-diet PKCε data, which were evaluated by unpaired non-parametric Mann–Whitney U test. *P<0.05, **P<0.01 and ***P<0.001 versus WT STD mice; ^$^*P*<0.05, ^$$^*P*<0.01 and ^$$$^*P*<0.001 versus *Apoe^−/−^* STD mice.

One of the main features of NAFLD is hepatic insulin resistance (González-Muniesa et al., 2017). For this reason, the hepatic expression of proteins involved in insulin signaling, such as insulin receptor β subunit (IRβ; also known as INSR), PI3 kinase p85α (p85α; also known as PIK3R1), p70 S6 kinase (p70S6K; also known as RPS6KB2) and protein kinase C epsilon type (PKCε; also known as PRKCE), was analyzed by western blotting (Fig. 4A,B). The expression of IRβ and p85α was markedly changed in the HFD-fed *Apoe^−/−^* mice, in which expression was significantly higher than in the STD-fed groups after 8 weeks on the diet but significantly lower after 18 weeks on the diet. IRβ expression was only significantly higher in STD-fed *Apoe*^−/−^ mice compared with STD-fed WT mice after 8 weeks on the diet (Fig. 4A,B). p70S6K expression in HFD-fed *Apoe*^−/−^ mice had a similar pattern to that of IRβ and p85α; however, in STD-fed *Apoe*^−/−^ mice, p70S6K expression was significantly lower than that in STD-fed WT mice after 8 weeks on the diet and significantly higher after 18 weeks on the diet (Fig. 4A,B). The expression of PKCε in STD-fed *Apoe*^−/−^ mice was similar after 8 and 18 weeks on the diet and significantly lower than that in STD-fed WT mice. In HFD-fed *Apoe*^−/−^ mice, PKCε expression was significantly higher than that in STD-fed *Apoe*^−/−^ mice and similar to that in STD-fed WT mice after 8 weeks on the diet, but significantly lower than that in WT mice after 18 weeks on the diet (Fig. 4A,B).

To evaluate glucose homeostasis, insulin tolerance tests (ITTs) and glucose intolerance tests (GTTs) were performed 17 weeks after weaning (Fig. 4C; Fig. S3). Our results suggested that, 17 weeks after HFD administration, mice developed overt and maintained glucose intolerance and insulin resistance.

Additionally, with the aim of analyzing glucose homeostasis alterations in our experimental conditions, *in vivo* insulin signaling studies were performed using 18-week STD- and HFD-fed *Apoe^−/−^* mice. AKT phosphorylation (Ser473 and Thr308) was also determined in response to insulin in the liver. Our results showed that insulin resistance exhibited by HFD-fed *Apoe^−/−^* mice in the ITT experiments could be due to an impairment in AKT phosphorylation (Thr308) because we did not observe an increase in the phosphorylation of this residue after insulin stimulation, hindering the full activation of this kinase (Fig. 4D).

### Role of autophagy in NAFLD development

**Fig. 5.**
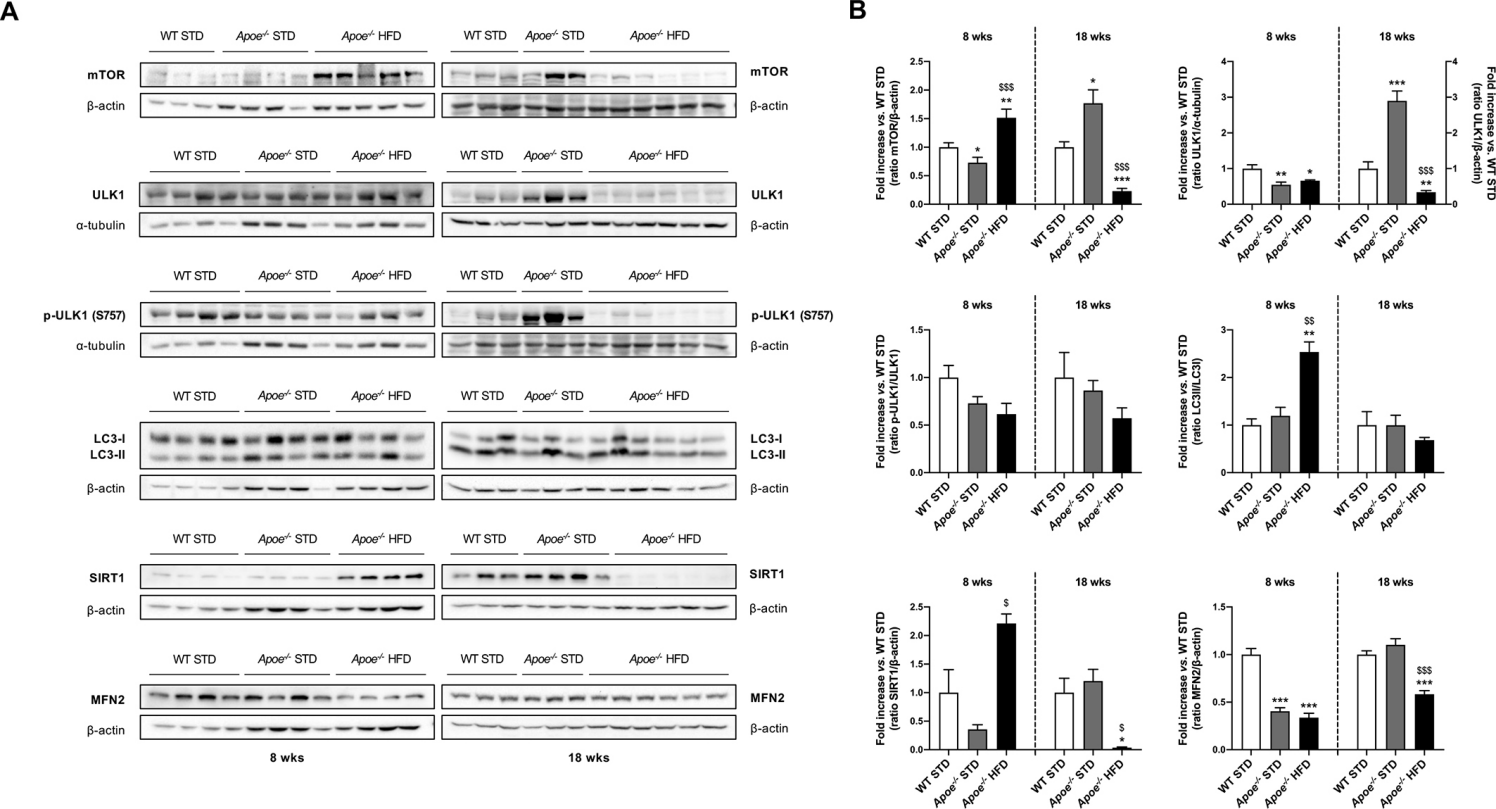
**Dysregulation of autophagy-related proteins in response to lipid oversupply.** (A) Representative gels of western blot analysis of mTOR, phospho-ULK1 (p-ULK1; Ser757), ULK1, LC3-I/II, SIRT1 and MFN2 in liver homogenates from the six groups studied (*n*=3-6 per group). α-tubulin or β-actin was used as a loading control. (B) Histograms showing the protein/α-tubulin or β-actin, p-ULK1/ULK1 and LC3-II/LC3-I ratio quantification of band intensities in WT STD (*n*=3-4), *Apoe^−/−^* STD (*n*=3-4) and *Apoe^−/−^* HFD (*n*=4-6) mice after 8 or 18 weeks on the diet. Results are expressed as mean±s.e.m. Statistical significance was assessed by two-tailed unpaired Student's *t*-test, with the exception of p-ULK1/ULK1 18-week and mTOR and SIRT1 8- and 18-week data, which were evaluated by unpaired non-parametric Mann–Whitney U test. *P<0.05, **P<0.01 and ***P<0.001 versus WT STD mice; ^$^*P*<0.05, ^$$^*P*<0.01 and ^$$$^*P*<0.001 versus *Apoe^−/−^* STD mice.

Although autophagy has emerged as a regulatory pathway, its activation state in the development of NAFLD remains unclear. In order to elucidate expression changes in NAFLD, we assessed the protein levels of mammalian target of rapamycin (mTOR), Unc-51-like autophagy-activating kinase 1 (ULK1) and its phosphorylated state, light chain 3 (LC3; also known as MAP1LC3), sirtuin 1 (SIRT1) and mitofusin 2 (MFN2) in liver samples by western blotting (Fig. 5A,B). mTOR expression in the *Apoe^−/−^* groups notably differed at 8 and 18 weeks. Although after 8 weeks on the diet, the expression of this protein was significantly decreased in STD-fed *Apoe^−/−^* mice compared with STD-fed WT mice, the opposite was observed after 18 weeks of feeding. In the HFD-fed *Apoe^−/−^* mice, mTOR expression was significantly higher than that in both STD-fed groups after 8 weeks on the diet and significantly lower after 18 weeks on the diet (Fig. 5A,B). A direct target of mTOR is ULK1, a protein involved in autophagy; its expression presented a similar pattern to that of mTOR, especially after 18 weeks of diet. Quantification showed a statistically significant reduction in ULK1 levels in both *Apoe^−/−^* groups compared with the WT group after 8 weeks on the respective diets. We observed that expression of this protein was similar in *Apoe^−/−^* HFD mice after 8 and 18 weeks on the diet, but significantly different from that of the STD-fed *Apoe^−/−^* mice. In the 18-week STD-fed *Apoe^−/−^* group, ULK1 expression was markedly increased compared with that at 8 weeks, and significantly higher than that in the STD-fed WT mice. Regarding the p-ULK1/ULK1 ratio, we observed lower levels in both *Apoe^−/−^* groups compared with WT, but these differences were not statistically significant (Fig. 5A,B). LC3 is a molecular marker of autophagy, which, once lipidated with phosphatidylethanolamine, triggers autophagosome formation, as seems to occur in 8-week HFD-fed *Apoe^−/−^* mice compared with STD-fed WT mice. Despite western blot analysis results indicating no significant differences between the groups, the data showed that HFD decreased the LC3-II/LC3-I ratio after 18 weeks of diet, suggesting the onset of autophagic flux restriction (Fig. 5A,B). The deacetylase SIRT1 has a key role in the regulation of autophagy by nutrients. Western blot analysis suggested that the higher levels of SIRT1 expression observed in the 8-week HFD-fed *Apoe^−/−^* mice compared with the STD-fed mice were only statistically significant with respect to the STD-fed *Apoe^−/−^* group, and that expression was significantly lower in this group than in the STD-fed groups after 18 weeks of feeding. In the STD-fed *Apoe^−/−^* group, SIRT1 expression was lower than that in the WT group after 8 weeks of diet, but almost the same after 18 weeks (Fig. 5A,B). The mitochondrial outer membrane protein MFN2 showed decreased expression in HFD-fed *Apoe^−/−^* mice at 8 and 18 weeks, exhibiting statistically significant differences with respect to STD-fed WT mice and, after 18 weeks of feeding, compared with STD-fed *Apoe^−/−^* mice. The STD-fed *Apoe^−/−^* group had decreased MFN2 levels after 8 weeks on the diet but the same levels as STD-fed WT mice after 18 weeks on the diet (Fig. 5A,B).

### Hepatic alteration in the expression of NAFLD-related miRNAs

Once we had characterized the experimental model, our next objective was to unravel the existing differences in NAFLD-related miRNA expression among the studied groups by RT-qPCR, using miR-191-5p as a normalizer.

**Fig. 6.**
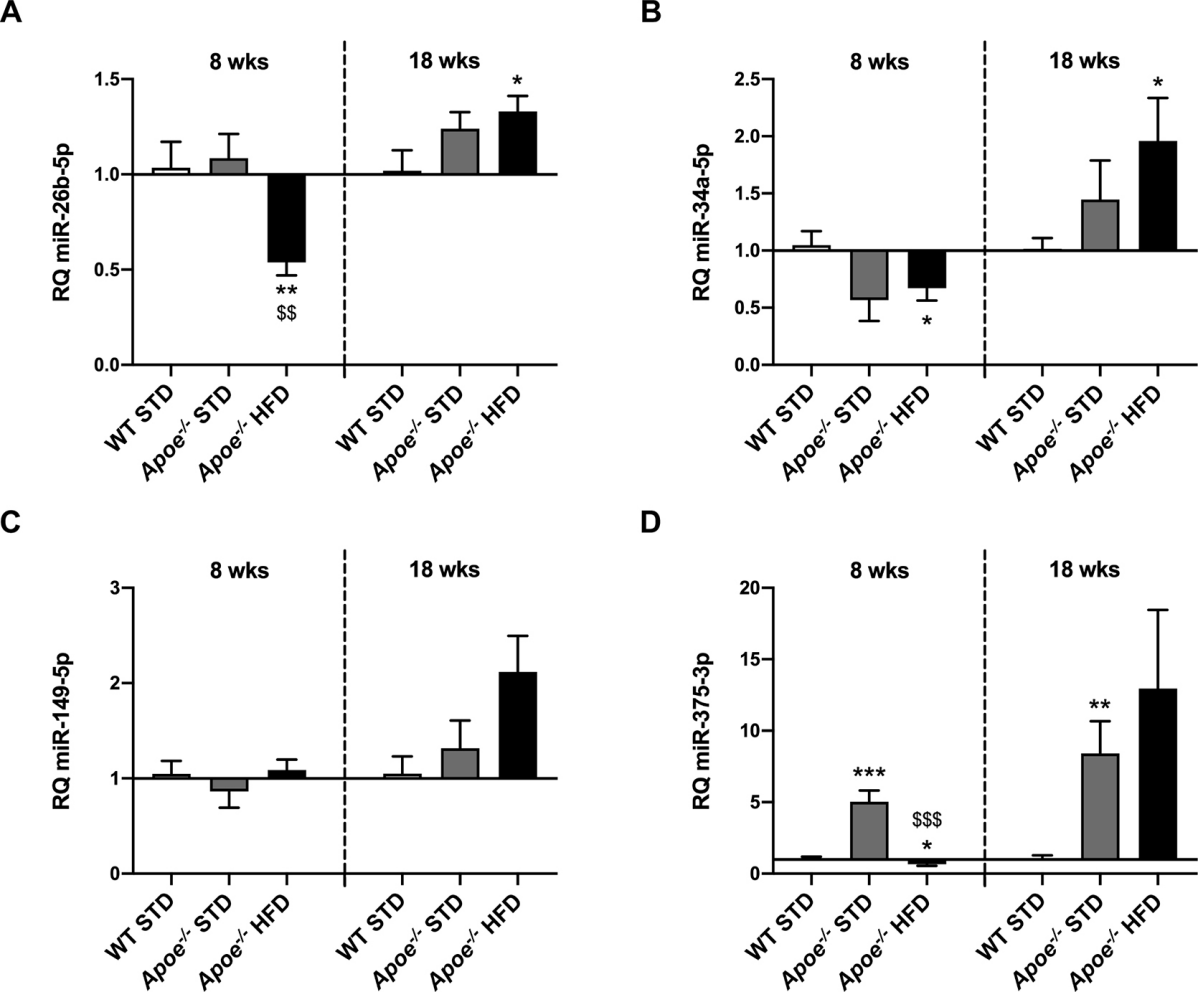
**RT-qPCR analysis of miRNA expression in the liver.** (A-D) Comparison of miR-26b-5p (A), miR-34a-5p (B), miR-149-5p (C) and miR-375-3p (D) expression in WT STD (*n*=4-7), *Apoe^−/−^* STD (*n*=4-9) and *Apoe^−/−^* HFD (*n*=6-10) mice after 8 or 18 weeks on the diet. miR-191-5p was used as a control. Results are expressed as mean±s.e.m. Statistical significance was assessed by two-tailed unpaired Student's *t*-test, with the exception of 18-week miR-149-5p data, which were evaluated by unpaired non-parametric Mann–Whitney U test. *P<0.05, **P<0.01 and ***P<0.001 versus WT STD mice; ^$$^*P*<0.01 and ^$$$^*P*<0.001 versus *Apoe^−/−^* STD mice.

First, in *Apoe^−/−^* mice fed a HFD for 8 weeks, we observed significantly lower levels of let-7d-5p (Fig. S4A), miR-22-3p (Fig. S4C), miR-26b-5p (Fig. 6A), miR-122-5p (Fig. S4E), miR-146b-5p (Fig. S4F), miR-181b-5p (Fig. S4G) and miR-194-5p (Fig. S4I) compared with those in STD-fed mice. The profile observed for miR-192-5p was rather similar, but levels in the STD-fed *Apoe^−/−^* mice were also significantly lower than those in STD-fed WT mice (Fig. S4H). Levels of miR-27b-3p and miR-34a-5p in HFD-fed *Apoe^−/−^* mice were significantly lower than those in STD-fed WT mice (Fig. 6B; Fig. S4D). In addition, significantly higher miR-375-3p levels were observed in STD-fed *Apoe^−/−^* mice compared with STD-fed WT mice, and significantly lower levels were observed in HFD-fed *Apoe^−/−^* mice compared with both STD-fed groups (Fig. 6D). Additionally, no statistical variation was observed in the levels of miR-15b-5p and miR-149-5p (Fig. 6C; Fig. S4B).

Regarding the 18-week-fed mice, levels of let-7d-5p, miR-22-3p and miR-194-5p tended to be lower, although not significantly, in the *Apoe^−/−^* groups than in the WT group (Fig. S4A,C,I). By contrast, miR-149-5p levels were higher, although not significantly, in *Apoe^−/−^* HFD mice than in the STD-fed mice (Fig. 6C). In the case of miR-375-3p, expression levels were higher in the two *Apoe^−/−^* groups than in the WT group, but a statistically significant difference was only observed between the STD-fed groups (Fig. 6D). Additionally, levels of miR-27b-3p expression were higher in the two *Apoe^−/−^* groups than in the WT group, but statistically significant differences were only obtained for the STD-fed *Apoe^−/−^* group compared with the WT group (Fig. S4D). For miR-26b-5p and miR-34a-5p, levels of expression were higher in the two *Apoe^−/−^* groups than in the WT group, significantly so in the case of HFD-fed *Apoe^−/−^* mice compared with STD-fed WT mice (Fig. 6A,B). The well-known hepato-specific miRNA miR-122-5p showed significantly higher expression in STD-fed *Apoe^−/−^* mice than in HFD-fed *Apoe^−/−^* and STD-fed WT mice (Fig. S4E). Although miR-146b-5p levels tended to be higher in STD-fed *Apoe^−/−^* mice and lower in *Apoe^−/−^* HFD mice than in STD-fed WT mice, these differences were not statistically significant (Fig. S4F). No significant differences in miR-15b-5p, miR-181b-5p and miR-192-5p expression were observed among the groups (Fig. S4B,G,H).

### Overexpression of miR-26b-5p and miR-34a-5p in Huh7 cells results in downregulated expression of some predicted targets

To demonstrate whether some selected miRNAs, such as miR-26b-5p and miR-34a-5p, are involved in regulating the expression of predicted targets, we transfected Huh7 cells with miR-26b-5p and miR-34a-5p miRNA precursors.

We observed that miR-34a-5p overexpression downregulates FAS, MFN2 and mTOR protein levels. In addition, miR-34a-5p transfection was validated by assessing the downregulation of SIRT1, a widely known target of this miRNA (Fig. S5).

We performed similar experiments overexpressing miR-26b-5p in order to validate our conclusions. In these experiments, we found a significant decrease in FAS expression (Fig. S5).

### EV miRNAs as potential NAFLD biomarkers

**Fig. 7.**
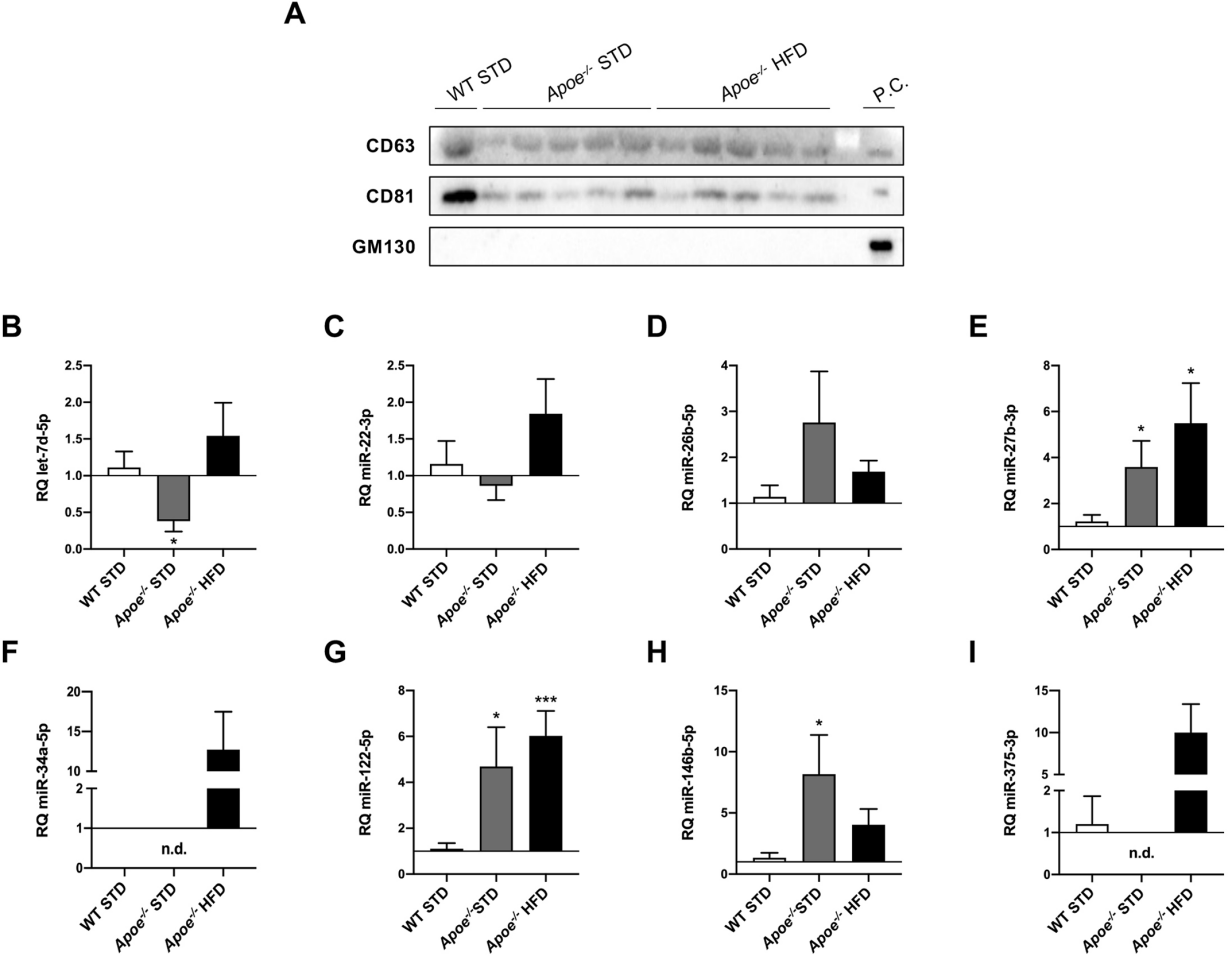
**RT-qPCR analysis of miRNA expression in circulating extracellular vesicles.** (A) Western blot analysis of markers to identify exosomes, such as CD63 and CD81, and GM130 as a *cis*-Golgi matrix protein used as a negative control, in WT STD (*n*=1), *Apoe^−/−^* STD (*n*=5) and *Apoe^−/−^* HFD (*n*=5) mice after 18 weeks on the diet. Huh7 cells were used as a positive control. (B-I) Comparison of let-7d-5p (B), miR-22-3p (C), miR-26b-5p (D), miR-27b-3p (E), miR-34a-5p (F), miR-122-5p (G), miR-146b-5p (H) and miR-375-3p (I) expression in WT STD (*n*=4-7), *Apoe^−/−^* STD (*n*=3-6) and *Apoe^−/−^* HFD (*n*=4-7) mice after 18 weeks on the diet. miR-16-5p was used as a control. Results are expressed as mean±s.e.m. Statistical significance was assessed by two-tailed unpaired Student's *t*-test, with the exception of miR-22-3p data, which were evaluated by unpaired non-parametric Mann–Whitney U test. *P<0.05 and ***P<0.001 versus WT STD mice. No statistics could be performed for miR-34a-5p and miR-375-3p data owing to undetermined values obtained in most WT STD and *Apoe^−/−^* STD liver samples. n.d., non-detected; P.C., positive control.

After demonstrating liver injury generated in the *Apoe^−/−^* model, we proceeded to isolate miRNAs contained in plasma EVs from 18-week-fed mice in order to examine, by RT-qPCR, whether some of the previous selected miRNAs were secreted into EVs and could be used as biomarkers of NAFLD. To validate EV isolation, CD63 and CD81, two of the most frequently used markers of exosomes, were analyzed by western blotting. GM130 (also known as GOLGA2) was used as a negative control because it is a *cis*-Golgi matrix protein. Our results suggest that the isolated EVs were enriched in exosomes (Fig. 7A).

The STD-fed *Apoe^−/−^* mice had significantly lower levels of let-7d-5p in plasma EVs compared with the STD-fed WT group (Fig. 7B). miR-22-3p contained in EVs tended to be higher, although not significantly, in HFD-fed *Apoe^−/−^* mice compared with the STD-fed group (Fig. 7C). The EV-contained miR-26b-5p levels tended to be higher, although not significantly, in the STD-fed *Apoe^−/−^* mice compared with the other groups (Fig. 7D). The content of miR-27b-3p and miR-122-5p in EVs was significantly higher in *Apoe^−/−^* mice, particularly HFD-fed *Apoe^−/−^* mice, than in STD-fed WT mice (Fig. 7E,G). The plasma miR-34a-5p and miR-375-3p levels presented similar profiles. In both cases, we observed a tendency for higher levels, although not significantly, in the HFD-fed *Apoe^−/−^* mice compared with the STD-fed groups and undetectable levels in the STD-fed *Apoe^−/−^* group (Fig. 7F,I). In 18-week-fed mice, EV-contained miR-146b-5p levels were higher in *Apoe^−/−^* mice than in WT mice, significantly so in the case of the STD-fed *Apoe^−/−^* group (Fig. 7H).

## DISCUSSION

Although the *Apoe^−/−^* murine model has been widely used in atherosclerosis research, in this study, we confirmed that these mice are also prone to developing hepatic steatosis and serve as a model for NAFLD, mimicking the major characteristics of this disease, as has been reported (Schierwagen et al., 2015; Hansen et al., 2017; Nascimento et al., 2020).

For instance, the abnormal amount of lipids in the liver, especially in HFD-fed mice, was caused by lipid level dysregulation as a consequence of insulin resistance, which is probably due to a defect in mitochondrial lipid oxidation (Saltiel and Kahn, 2001; Stumvoll et al., 2005; Muoio and Newgard, 2008; Castro et al., 2014). From a clinical perspective, if we consider the results obtained after the double-blind hepatic examination, it could be said that HFD administration accelerated disease progression without changes in food intake between the groups. The lipid droplet size itself represents an essential parameter indicating the processes used for cellular TG catabolism, especially under nutrient oversupply. Two TG disposal pathways exist in hepatocytes, lipolysis and lipophagy, which degrade large and small lipid droplets, respectively (Schott et al., 2019). Although NAS did not differ between the two experimental *Apoe^−/−^* HFD groups, lipid droplets were obviously bigger after 18 weeks of feeding. Furthermore, the hepatic fibrosis increased with phenotype severity. Thus, the observed fibrosis was in a preliminary stage.

Intriguingly, *Cd36* expression seemed to be highest in STD-fed *Apoe^−/−^* mouse livers. Although CD36 is weakly expressed in hepatocytes, it makes sense that accumulated lipid overload upregulates its expression, reinforcing clinical studies that have described a CD36 content increase in NAFLD patients (García-Monzón et al., 2014; Rada et al., 2020). However, *Cd36* mRNA levels in HFD-fed *Apoe^−/−^* mice were not augmented as expected but were similar to those in STD-fed WT mice. It should be pointed out that *Cd36* deletion increases *Ccl2* transcription in hepatocytes without affecting hepatic lipid uptake (Zhong et al., 2017). The chemokine CCL2 is produced in adipose tissue and triggers the development of insulin resistance and steatosis (Xu et al., 2015), two of the major characteristics present in the HFD-fed *Apoe^−/−^* mice, in which its expression was significantly increased. Therefore, *Cd36* deficiency might contribute to the progression of non-alcoholic fatty liver to NASH, which is managed by inflammation and subsequent cytokine and chemokine production.

ACC catalyzes the rate-limiting step of *de novo* lipogenesis and regulates fatty acid β-oxidation in hepatocytes. In our mouse model, the HFD-fed *Apoe^−/−^* group showed dramatically reduced ACC expression. Many reports have demonstrated that a decrease in *Acaca* expression indicates steatosis amelioration (Donnelly et al., 2005; Li et al., 2018; Lopez-Pastor et al., 2019). However, in accordance with our findings, Chow et al. observed that the phenotype of their liver-specific ACC1/2 double knockout mice promoted hepatic TG accumulation and fatty acid oxidation repression, thus preserving fat storage in this tissue by a compensatory pathway (Chow et al., 2014). Liver SCD1 protein levels were significantly higher in HFD-fed *Apoe^−/−^* mice than in STD-fed mice after 8 weeks on the diet, but significantly lower after 18 weeks. Previous reports suggested that SCD1 downregulation aggravates hepatocyte lipoapoptosis and liver injury (Cazanave and Gores, 2010; Fernández Gianotti et al., 2013). Two HFD-fed rat models displayed a similar downregulation of *Scd1* mRNA and protein levels, suggesting that SCD oligomer formation can alter enzyme activity and function, thus aggravating NAFLD (Fernández Gianotti et al., 2013). Regarding the global downregulation of the lipogenic enzymes in our 18-week HFD-fed *Apoe^−/−^* mice, Luo et al. reported that these effects can be related to high-fat feeding (Luo et al., 2012). After feeding, SREBP1c is activated by insulin signaling, upregulating lipogenic genes. However, our HFD-fed mice presented insulin resistance, avoiding this upregulation. A reduction in SREBP1c and, subsequently, lipogenic genes was described in liver-specific insulin receptor knockout mice, in agreement with our findings (Haas et al., 2012).

A pivotal and common feature of metabolic disease is insulin resistance, and the overwhelming effect of a HFD has been associated with increased prevalence of this impairment, as our outcomes show. In the liver, the suppression of glucose production and stimulation of lipogenesis and glycogen synthesis are insulin-mediated processes, the dysregulation of which affects both insulin secretion and action, producing elevation in fasting and postprandial glucose and lipid levels (Michael et al., 2000; Saltiel and Kahn, 2001). Regarding this, although increased expression of IRβ was found in 8-week HFD-fed *Apoe^−/−^* mouse livers, suggesting an initial compensatory mechanism against HFD-induced insulin resistance, this increase was no longer observed after 18 weeks on the diet. A study based on partial deletion of hepatic IR in mice fed a HFD observed an amelioration of hepatic steatosis (Merry et al., 2020); therefore, the decrease observed in our HFD-fed *Apoe^−/−^* mice could serve as an attempt to avoid NAFLD progression. Indeed, under pathophysiological and nutrient excess conditions, the induction of endoplasmic reticulum stress degrades IR by autophagy-dependent processes, contributing to obesity-induced insulin resistance in peripheral tissues (Zhou et al., 2009). In the same way, we also observed a reduction in several proteins involved in insulin signaling in 18-week HFD-fed mice, contributing towards insulin resistance. Upon hepatic insulin resistance, the content of diacylglycerol (DAG) leads to PKCε activation, which in turn plays a major role in binding to the IR and repressing its tyrosine kinase activity by Thr1160 phosphorylation (Monetti et al., 2007; Jornayvaz et al., 2011; Petersen et al., 2016; Lyu et al., 2020). Certainly, our findings revealed decreased PKCε expression, but this did not correlate with a reduction in insulin resistance. In a previous study, mice with *Prkce* ablation and 16 weeks of fat feeding did not exhibit reversed diet-induced inhibition of insulin signaling in the liver; thereby, PKCε-independent mechanisms might maintain long-term insulin resistance (Raddatz et al., 2011). Bearing in mind *in vivo* insulin signaling studies and the aforementioned insulin resistance observations, western blot analysis of AKT activation failed to show statistically significant differences between the 18-week STD- and HFD-fed *Apoe^−/−^* groups, verifying insulin signaling impairment. As has previously been described, insulin-induced AKT activation requires phosphorylation of both Ser473 and Thr308 sites to result in synergistic and full activation, and these phosphorylations are not dependent on each other (Alessi et al., 1996; Taniguchi et al., 2006).

As seen throughout the study and as NAFLD progresses, abrupt changes occur in the expression of different proteins involved in either glucose or lipid metabolism. These changes can be triggered by many mediators, including miRNAs. Moreover, these molecules are recognized as potential biomarkers of NAFLD. Indeed, comparing NAFLD patients and healthy individuals, miR-375 is upregulated in pathologic serum samples (Pirola et al., 2015; Lei et al., 2018). Although miR-375 function in NAFLD has still not been elucidated, our results revealed a noteworthy increase in 18-week HFD-fed *Apoe^−/−^* mice and *Adipor2* downregulation, as previously reported (Lei et al., 2018). This result is of importance as *Adipor2* acts as a regulator of glucose and lipid metabolism through modulating the peroxisome proliferator-activated receptor alpha (PPARα) pathway (Lei et al., 2018). As other authors have checked *in vivo* and *in vitro* (Choi et al., 2013; Fu and Kemper, 2016; Xu et al., 2021b), another miRNA that regulates this gene is miR-34a, which is also overexpressed in our model. This miRNA is altered in NAFLD and other related diseases, such as obesity and atherosclerosis. Its expression is triggered by lipids and aggravates NAFLD and NASH, either by impairing lipid accumulation, promoting oxidative stress or fostering hepatocyte apoptosis (Lee et al., 2010; Castro et al., 2013; Choi et al., 2013; Zhang et al., 2020; Xu et al., 2021a,b). The regulation of *Adipor2* by miR-34a induces ectopic deposition of lipids and decreases mitochondrial content by resistin action through the AMP-activated protein kinase (AMPK)/PPARα pathway (Wen et al., 2018). These findings and our database research suggest that *Adipor2* expression can be modulated by miR-149-5p. The prominent hepatic increase in *Ppargc1a* mRNA in the HFD-fed group might be due to a compensatory response to enhance mitochondrial biogenesis and function in a steatosis environment, foster fatty acid oxidation, and reduce TG accumulation and secretion (Morris et al., 2012). Furthermore, it has been described that miR-34a decreases expression of SREBP1c as well as that of its downstream targets, ACC, FAS and SCD1 (Wang et al., 2020), which would explain our findings based on their downregulated expression after 18 weeks of HFD feeding. In the same way, our results suggest that overexpression of miR-34a-5p in cultured hepatocytes induced evident FAS downregulation. Database analysis revealed that these three enzymes were not only targets of miR-34a-5p, but also miR-149-5p, as our results confirmed. Although the role of miR-149 in the liver remains unclear, it is elevated in a HFD-induced NAFLD mouse model and fibroblast growth factor-21 (FGF-21) was identified as its target (Xiao et al., 2016; Chen et al., 2020). In addition, miRNA–target interaction (MTI) databases predict *Fasn* as a miR-26b-5p and miR-375-3p target, which is consistent with the outcomes obtained after 18 weeks of feeding and with our *in vitro* experiments on miR-26b-5p overexpression. Concerning insulin signaling, IR and p85α are targets of some assessed miRNAs, but, according to our data, only miR-149-5p or miR-375-3p regulation would explain the decrease in expression observed after 18 weeks on the HFD and, as a consequence, the insulin resistance. Indeed, miR-149-5p was upregulated in the liver of HFD-induced hepatic insulin resistance mice (Zhao et al., 2019), and miR-375 is highly expressed in pancreatic islets involved in insulin secretion and glucose homeostasis (Poy et al., 2004, 2009). Thus, according to our results and database research, p85α expression can also be regulated by miR-34a-5p, while PKCε is a target of miR-34a-5p and miR-375-3p. Moreover, p70S6K might be regulated by miR-26b-5p and miR-34a-5p in 18-week HFD-fed *Apoe^−/−^* mice.

Autophagy is a quality control program that can not only degrade abnormal subcellular organelles and misfolded proteins, but also lipid droplets, especially in the early stage of steatosis, alleviating disease progression. However, its inhibition occurs in the late stage of NAFLD progression (Ding et al., 2020; Lee et al., 2020; Lu et al., 2020). For this reason, although research on autophagy in NAFLD is still scarce, evidence suggests that its dysfunction is closely related to this liver disease. Consistent with what is mentioned above, our results indicated increased LC3 lipidation in 8-week HFD-fed *Apoe^−/−^* mice and, therefore, autophagic flux activation as observed in the early stages of NAFLD. However, after 18 weeks of feeding, ULK1 expression and LC3 lipidation were decreased in the livers from HFD-fed *Apoe^−/−^* mice, suggesting autophagy inhibition, a feature previously described in advanced stages of NAFLD (Ding et al., 2020; Lee et al., 2020; Lu et al., 2020). Lee et al. attributed this phenomenon to long-term HFD feeding (Lee et al., 2020). Similar outcomes were obtained for SIRT1 expression, another regulator of autophagy through autophagy-related (ATG) protein modulation. Our findings showed a reduction in SIRT1 expression after 18 weeks of HFD feeding, as occurred in a HFD-induced NAFLD rat model (Deng et al., 2007). In liver diseases, mitochondrial dysfunction triggers oxidative stress, inflammation and lipotoxicity. Activation of the SIRT1/PGC-1α pathway effectively induces mitophagy, which is involved in mitochondrial dysfunction resolution (Jiang et al., 2021). Aside from autophagy, liver-specific SIRT1 knockout mice challenged with HFD developed hepatic steatosis, inflammation and endoplasmic reticulum stress. As a consequence of SIRT1 loss, PPARα signaling failed, decreasing fatty acid oxidation and PPARα target gene expression, including *Cd36* expression, as our aforementioned findings show (Purushotham et al., 2009). Another hallmark of NAFLD is morphological alterations of mitochondria. Mitofusin proteins are located at the outer mitochondrial membrane and control fusion events, maintaining a proper energetic and metabolic cellular performance. In particular, MFN2 has a direct role in lipid transfer, especially phosphatidylserine, between mitochondria and endoplasmic reticulum, prompting progression of liver diseases if depleted, as our findings in *Apoe^−/−^* HFD mice showed (Hernández-Alvarez et al., 2019; Joaquim and Escobar-Henriques, 2020). Another consequence of a decrease in MFN2 is impairment of glucose, pyruvate and fatty acid oxidation. Under excessive lipid intake, this loss is also connected to mitophagy defects, leading to the accumulation of dysfunctional mitochondria, unmetabolized fatty acid overload in mitochondria and, ultimately, steatosis (Joaquim and Escobar-Henriques, 2020).

Next, we assessed the role of miRNAs in the regulation of proteins involved in autophagy. MTI databases predict that miR-34a-5p could be targeting mTOR and ULK1 expression, which is aligned with our results. It is also likely that autophagy regulation occurs elsewhere in the signaling pathway, owing to long-term presence of nutrients. After 8 weeks of HFD administration, our results revealed upregulated SIRT1, which has been reported as beneficial upon HFD exposure. The effects described in a previous study were the prevention of hepatosteatosis targeting SREBP1c, the induction of antioxidant enzymes, the protection against inflammation and diet-induced glucose intolerance (Pfluger et al., 2008). The upregulation and later downregulation of SIRT1 expression found in our HFD mouse model are consistent with miR-34a-5p expression differences, and several works have described the deacetylase as a miR-34a-5p target (Ding et al., 2015; Wang et al., 2020). Indeed, Choi et al. also reported that the elevation of hepatic miR-34a is related to the severity of NAFLD and reduced SIRT1 in the liver on account of targeting nicotinamide phosphoribosyltransferase (NAMPT) and, consequently, impairing NAD biosynthesis (Choi et al., 2013). In addition to miR-34a-5p, we have found that SIRT1 is also a target of miR-26b-5p, and its protein expression might have corresponded with a synergistic effect, although miR-26b-5p has not yet been described as a NAFLD mediator, unlike its family member, miR-26a (Ali et al., 2018; Xu et al., 2021a,b). Finally, we hypothesized that the lower levels of MFN2 in HFD-fed *Apoe^−/−^* mice could be due to miR-149-5p and/or miR-34a-5p regulation. Although MTI databases predicted the regulation of MFN2 by miR-149-5p, this has not yet been described. Conversely, in palmitic acid-induced muscle cell dysfunction, miR-34a-5p overexpression downregulated MFN2 expression (Simão et al., 2019). We found the same outcome in our miR-34a-5p overexpression assays in cultured hepatocytes.

Recently, EVs have gained attention owing to their potential role in disease development as a mechanism of cell–cell communication. Within their cargoes, miRNAs have emerged as promising non-invasive biomarkers for the diagnosis of many diseases, including NAFLD (Povero et al., 2014; Tan et al., 2014; Garcia-Martinez et al., 2020; Srinivas et al., 2021). According to the literature and our data, miR-34a and miR-375 may be potential biomarkers of NAFLD due to the marked increase in expression observed in the plasma of HFD-fed mice, as has been previously described in the serum of NAFLD patients (Yamada et al., 2013; Pirola et al., 2015). Furthermore, the higher plasma levels of miR-27b-3p and miR-122-5p, the more severe the disease. Hence, these two circulating miRNAs might serve as NAFLD-stage markers, supporting previous publications (Yamada et al., 2013; Tan et al., 2014). Finally, we propose circulating let-7d-5p and miR-146b-5p as biomarkers of early stages of NAFLD because both are only significantly altered in the STD-fed *Apoe^−/−^* group.

In summary, NAFLD is a multifactorial complex disease with limited diagnostic and treatment tools, which threatens a large part of the world’s population. Increasing knowledge of miRNAs and their performance and/or regulation has allowed the investigation of the mechanisms involved in obesity-associated metabolic diseases, including NAFLD. Considering the research on miRNA expression levels and MTI databases in NAFLD mice administered a HFD, we suggest that decreases in lipogenesis-related proteins, such as ACC, FAS and SCD1, are due to miR-34a-5p and miR-149-5p regulation. In relation to insulin resistance, they could be modulated by miR-34a-5p, miR-149-5p and miR-375-3p, targeting IR and p85α. We have also confirmed the pivotal role of autophagy in NAFLD, which is negatively impaired because miR-34a-5p induced a decrease in mTOR, ULK1, SIRT1 and MFN2 expression. Regarding circulating miRNAs, the initial stages of NAFLD might be diagnosed by measuring let-7d-5p and miR-146b-5p levels; the later stages are associated with notable increases in circulating miR-34a-5p and miR-375-3p levels. As disease progresses, miR-27b-3p and miR-122-5p plasma levels increase. The findings of this study have been summarized in Fig S7. Further investigation will be necessary to shed light on the interplay between miRNAs and their targets, as well as elucidate their participation in NAFLD progression and their potential use as biomarkers.

## MATERIALS AND METHODS

### Mice and diets

WT and *Apoe^−/−^* male mice, both of a C57BL/6J genetic background, were purchased from The Jackson Laboratory (Bar Harbor, ME, USA) and maintained on a 12 h light/dark cycle at room temperature. Animals were fed *ad libitum* with a STD (5.3% calories from fat; D.Rod18.H07, LASvendi GmbH, Soest, Germany) or HFD (60% calories from fat; TD 06414, Envigo Teklad, Barcelona, Spain) from weaning until euthanasia at 8 or 18 weeks. For each period of study, three groups (5-14 mice/group) were established: STD-fed WT, STD-fed *Apoe^−/−^* and HFD-fed *Apoe^−/−^*. Sacrifice of 16 h-fasted mice was performed after 8 or 18 weeks on the respective diets. All animal experimentation was conducted in accordance with the accepted standards of animal use approved by the Complutense University of Madrid Committee.

### *In vivo* insulin signaling studies

In order to study the hepatic insulin signaling in physiological conditions, *in vivo* insulin signaling assays were performed. Fasted mice were intraperitoneally injected with 1 U/kg BW of insulin glulisine (Apidra SoloStar, Sanofi, Paris, France). After 10 min, mice were sacrificed and harvested tissues were immediately frozen in liquid nitrogen. Insulin signaling was assessed by western blot analysis of phospho-AKT (Ser473 and Thr308) in liver homogenates.

### Metabolic tests

GTTs and ITTs were performed as we have previously described (Lopez-Pastor et al., 2019). Assays of total Ch and total TGs (Spinreact, Girona, Spain), as well as transaminase levels (Spinreact, Girona, Spain), were performed with plasma samples obtained from 16 h-fasted mice. According to Triglyceride Colorimetric Assay Kit (Cayman Chemical, Ann Arbor, MI, USA) instructions, intrahepatic TGs were measured in liver samples.

### EV extraction

Briefly, plasma was previously clarified by two sequential 20-min centrifugations at 2000 ***g*** and 10,000 ***g***. Then, EVs were pelleted with Total Exosome Precipitation Reagent (Invitrogen, Carlsbad, CA, USA). Pelleted vesicles were resuspended in Exosome Resuspension Buffer (Invitrogen) for further processing.

### Cell culture

Huh7 cells were kindly supplied by Dr González-Rodríguez (Instituto de Investigación Sanitaria Princesa, Madrid, Spain). They were maintained in 75 cm^2^ flasks (Thermo Fisher Scientific, Waltham, MA, USA) in high-glucose Dulbecco's modified Eagle medium (Cytiva, Marlborough, MA, USA) supplemented with 10% fetal bovine serum (Gibco, Waltham, MA, USA), 100 U/ml penicillin-streptomycin (Gibco) and MycoZap (Lonza, Basel, Switzerland).

### Transfection with miR-26b-5p and miR-34a-5p miRNA precursors

Huh7 cells were seeded in 9.6 cm^2^ six-well plates in complete medium (1×10^5^ cells per well). After 24 h, growth medium was replaced with fresh medium. Cells were then transfected with a miR-26b-5p and miR-34a-5p precursor (PM12899 and PM11030, respectively) (Ambion, Austin, TX, USA) using INTERFERin^®^ (Polyplus Transfection, Strasbourg, France) as the transfection reagent. Once transfection conditions were established, final concentrations in the wells were 10 nM or 20 nM for the miR-26b-5p precursor, and 5 nM or 10 nM for the miR-34a-5p precursor, in 1 ml final volume. After 6 h, cells were supplemented with an additional milliliter of complete medium. The medium was discarded 96 h after transfection and cells were washed with cold phosphate-buffered saline (PBS) (Gibco).

### PCR

*Apoe^−/−^* mice were genotyped by PCR with DNA AmpliGel Master Mix (Biotools, Madrid, Spain) as previously described (Gómez-Hernández et al., 2013). A 155-bp band was obtained for the WT mice and a 245-bp band for the *Apoe^−/−^* mice.

### Isolation of RNA and miRNAs

According to the manufacturer's protocol, RNA was isolated from liver tissue and cultured cells with a miRVana miRNA Isolation Kit (Invitrogen), and RNA extraction from EVs was carried out with a Total Exosome RNA & Protein Isolation Kit (Invitrogen). In both cases, miRNAs and long RNAs were obtained in separate fractions.

### RT-qPCR

Complementary DNA (cDNA) was synthesized by a High-Capacity cDNA Reverse Transcription Kit (Applied Biosystems, Foster City, CA, USA) for mRNA analysis. cDNA from miRNAs was obtained using a TaqMan^™^ Advanced miRNA cDNA Synthesis Kit (Applied Biosystems). Analysis of gene expression (*Acaca*, *Adipor2*, *Cd36*, *Fasn*, *Ppargc1a*, *Scd1*) was performed with the corresponding primers using PowerUp™ SYBR^®^ Green Master Mix (Applied Biosystems). Analysis of *Ccl2* and miRNA expression (let-7d-5p, miR-15b-5p, miR-22-3p, miR-26b-5p, miR-27b-3p, miR-34a-5p, miR-122-5p, miR-146b-5p, miR-149-5p, miR-181b-5p, miR-192-5p, miR-194-5p and miR-375-3p) was performed with TaqMan probes for the corresponding genes using TaqMan™ Fast Advanced Master Mix (Applied Biosystems). All RT-qPCR experiments were performed in an ABI Prism 7900HT Thermal Cycler (Applied Biosystems). The relative abundance of mRNAs or miRNAs was calculated using an endogenous reference gene (*Gapdh* for conventional TaqMan^®^, miR-191-5p for TaqMan^®^ from miRNAs, miR-16-5p for TaqMan^®^ from miRNAs of EVs, and *Actb* for SYBR^®^). SYBR Green primer sequences and TaqMan probes are shown in Tables S1 and S2, respectively. The results were calculated using the 2^−ΔΔCq^ method.

### Western blotting

Western blot analyses were performed on liver homogenates and cultured cells as previously described (Escribano et al., 2003). The primary antibodies used are shown in Table S3 and all of them were diluted in TTBS. Rabbit and mouse primary antibodies were immunodetected using horseradish peroxidase-conjugated anti-rabbit (NA931V; 1:4000 in TTBS) or anti-mouse secondary antibody (NA934V; 1:5000 in TTBS) (GE Healthcare, Buckinghamshire, UK), respectively. When possible, phospho-proteins and their total expression were detected in the same gel, using Restore^TM^ Western Blot Stripping Buffer (Thermo Fisher Scientific) as per the manufacturer's instructions, blocking the membrane again before the incubation with the other antibody. Loading was normalized by α-tubulin or β-actin. The representative gels that share the same housekeeper were as follows: (1) at 8 weeks, ACC and p70S6K; (2) at 8 weeks, p85α, SIRT1 and MFN2; (3) at 8 weeks, PKCε, ULK1 and p-ULK1; (4) at 18 weeks, ACC and p-ULK1; (5) at 18 weeks, FAS, IRβ, p85α and SIRT1; (6) at 18 weeks, SCD1 and LC3. The band intensities were quantified using ImageJ v1.52k software (http://rsb.info.nih.gov/ij).

### Histological characterization of hepatic lesions

A section of liver samples was included in Tissue-Tek^®^ optimum cutting temperature (OCT) compound (Sakura Finetek, Alphen aan den Rijn, The Netherlands), and later in liquid nitrogen for freezing, and another one was maintained in formalin to be included in paraffin. Each OCT-embedded liver block was serially sectioned (7 μm) with a cryostat (CM1510 S, Leica, Wetzlar, Germany) to perform the Oil Red O staining using standard techniques to quantify lipid accumulation. Paraffin-embedded livers were cross-sectioned into 4-μm-thick specimens at 5-mm intervals, dewaxed, and rehydrated to be stained with H&E to analyze the architecture or Sirius Red to measure the hepatic fibrosis. Images of sections of H&E, Sirius Red and Oil Red O were acquired using an inverted Eclipse TE300 microscope coupled to a Digital Sight DS-U2 camera (Nikon, Tokyo, Japan). Quantifications for images of Oil Red O staining were performed using IP Win32 v4.5 software (Acromag, Wixom, MI, USA). Lipid droplet size was quantified using ImageJ v1.52k software, and quantification for images of Sirius Red stains was carried out using Fiji software (NIH, Bethesda, MD, USA).

The assessment of NAS in liver samples was performed as we have previously described (Lopez-Pastor et al., 2019). Assessment of fibrosis stage was carried out by an experienced liver pathologist using the system for mouse models validated by Liang et al., which defined fibrosis staging as: 0, none; 1, perisinusoidal and/or pericentral; 2, incomplete central/central bridging fibrosis; 3, complete central/central bridging fibrosis (Liang et al., 2014).

### *In silico* analysis of miRNAs and targets involved in NAFLD

The identification of the different miRNAs that were analyzed in the study was carried out through an exhaustive search for keywords and different publications in PubMed. Once the miRNAs of interest were chosen, the interaction between them and their mRNA targets was evaluated by searching prediction databases such as miRWalk v3.0, TargetScan Mouse v7.2, miRDB v5.0 and miRTarBase v6.0. The data collected from these databases were analyzed, and only those targets that appeared in two or more databases were considered as possible targets. The choice was also made considering that these targets were key proteins in the signaling pathways that can be altered in NAFLD such as lipogenesis, insulin signaling and autophagy. A diagram showing the MTIs during NAFLD progression that are proposed throughout the main text is supplied in Fig. S6. This representation was made using Cytoscape 3.8.2. software (Shannon et al., 2003).

### Statistical analysis

Data are presented as mean±s.e.m. Normality of these variables was tested with Shapiro–Wilk test. Differences were assessed using unpaired two-tailed Student's *t*-tests and unpaired non-parametric Mann–Whitney U tests, as appropriate. *P*<0.05 was considered statistically significant. The software used for the analyses was Prism v8.0 (GraphPad Software Inc., San Diego, CA, USA).

## Supplementary Material

Supplementary information
