# Supplementary material for: Concerted regulation of non-alcoholic fatty liver disease progression by microRNAs in apolipoprotein E-deficient mice
Source: Dis Model Mech. 2021 Dec 24;14(12):dmm049173. doi: 10.1242/dmm.049173 (PMC8713993; doi:10.1242/dmm.049173)
Supplement: Supplementary information [file dmm-14-049173-s1.pdf]

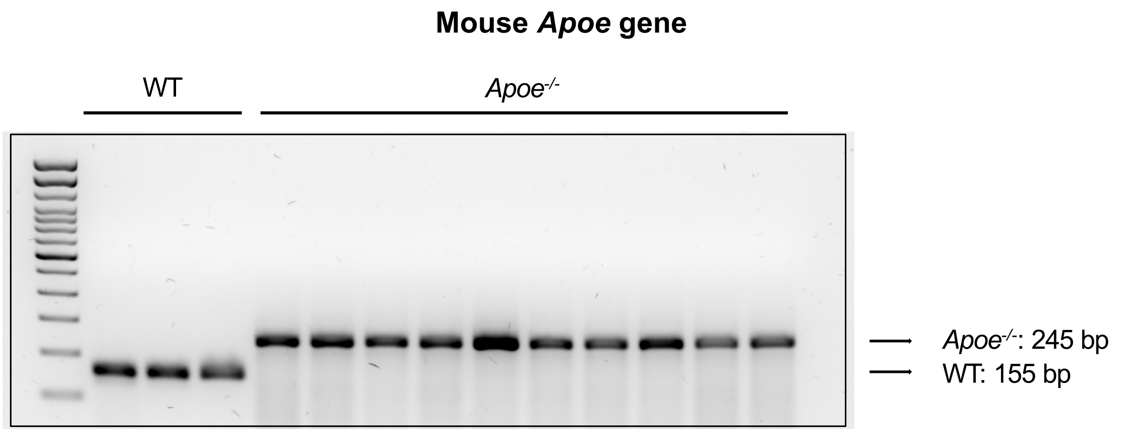

Fig. S1. Genotype assessment of the mouse model. DNA extracted from the tail of WT STD (n = 3) and *Apoe*$^{-/-}$ (n = 10) mice was used to perform the genetic characterization through PCR analysis. bp: Base pairs.

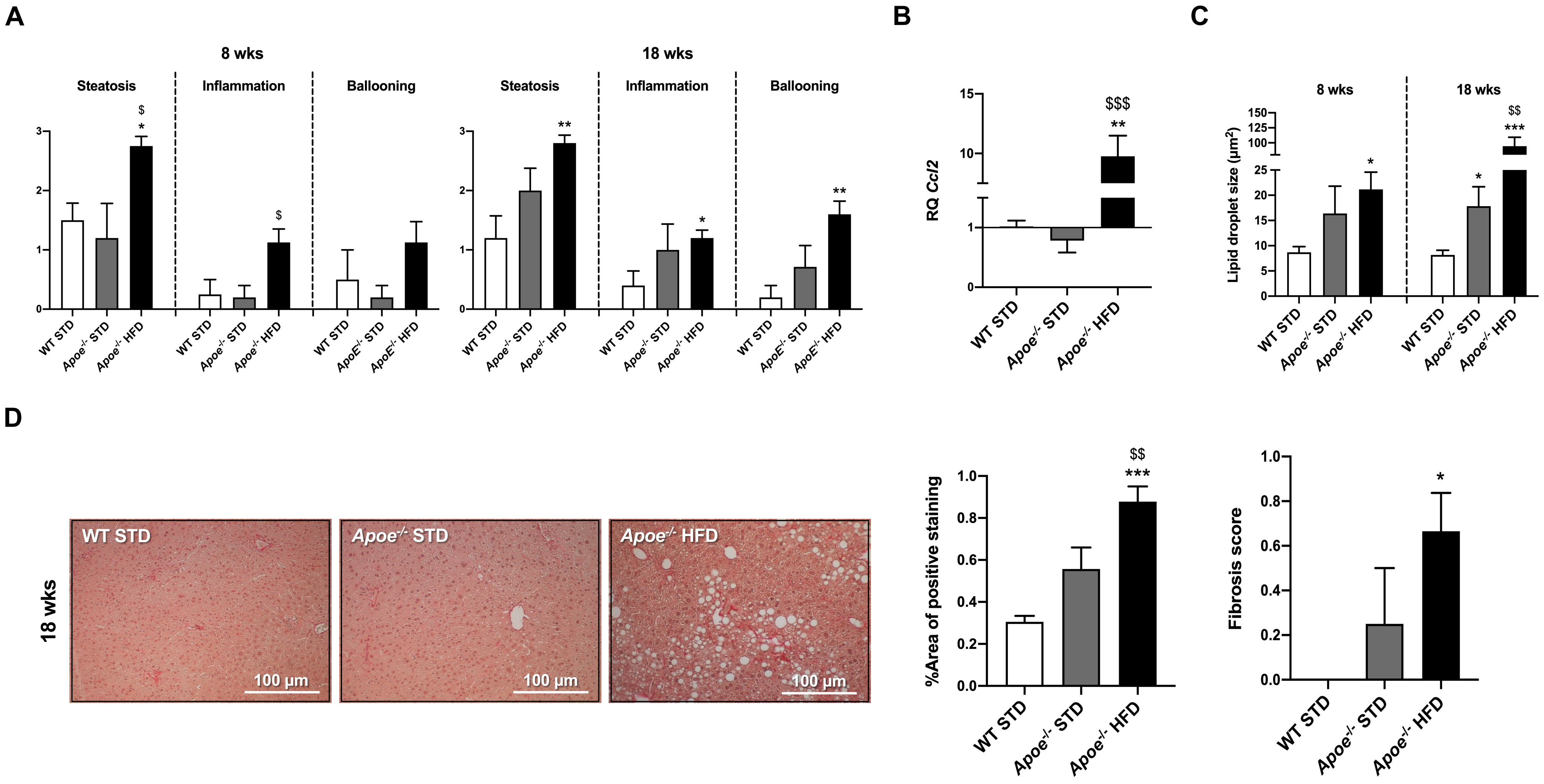

Fig. S2. **Histopathological analysis of NAFLD progression.** (A) The quantification of steatosis, inflammation and ballooning in WT STD (n = 4-5), *Apoe*$^{-/-}$ STD (n = 5-7) and *Apoe*$^{-/-}$ HFD (n = 8-10) mice after 8 (*left*) or 18 (*right*) weeks on diet. (B) mRNA levels of *Ccl2* by RT-qPCR in livers from WT STD (n = 5), *Apoe*$^{-/-}$ STD (n = 8) and *Apoe*$^{-/-}$ HFD (n = 11) mice fed on 18 weeks. Gapdh was used as a control. (C) Measurement of lipid droplet size (μm2) in WT STD (n = 5-6), *Apoe*$^{-/-}$ STD (n = 3-6) and *Apoe*$^{-/-}$ HFD (n = 8-9) mice fed for 8 or 18 weeks. (D) Representative images of Sirius Red staining to study hepatic fibrosis (image magnification: x10) and the quantification of positive area staining and fibrosis score in WT STD (n = 4), *Apoe*$^{-/-}$ STD (n = 4) and *Apoe*$^{-/-}$ HFD (n = 6-12) mice after 18 weeks on diet. Results are expressed as mean ± SEM. Statistical significance was assessed by two-tailed unpaired Student's *t*-test with the exception of Sirius Red positive area and fibrosis score data which were evaluated by unpaired non-parametric Mann-Whitney U test and it was represented as * ($p < 0.05$), ** ($p < 0.01$) and *** ($p < 0.001$) *vs.* WT STD mice; $ ($p < 0.05$), $$ ($p < 0.01$) and $$$ ($p < 0.001$) *vs. Apoe*$^{-/-}$ STD mice.

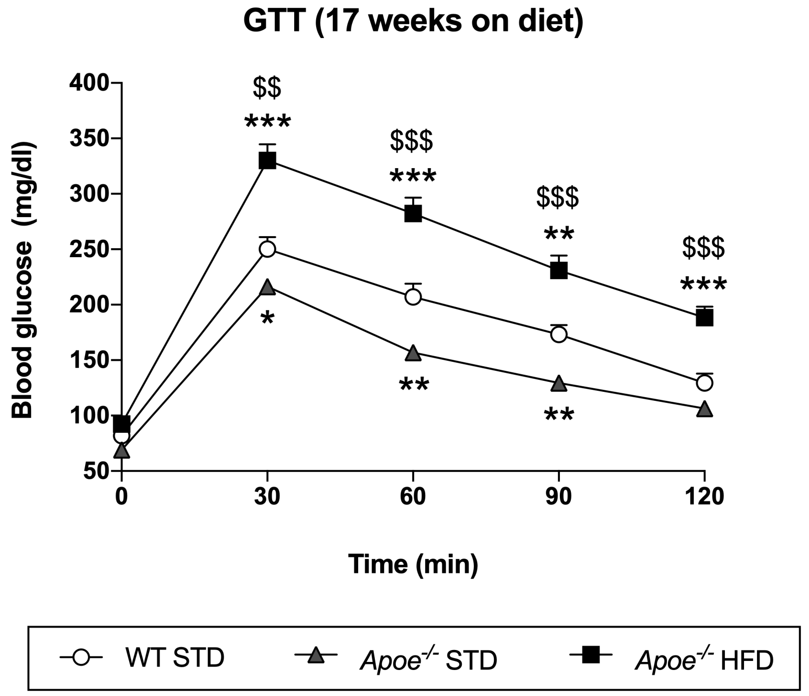

Fig. S3. Glucose tolerance is also altered in mice with liver injury. GTT in WT STD (n = 7), *Apoe*$^{-/-}$ STD (n = 5-6) and *Apoe*$^{-/-}$ HFD (n = 7) animals after 17 weeks on diet. Results are expressed as mean ± SEM. Statistical significance was assessed by two-tailed unpaired Student's *t*-test and it was represented as * ($p < 0.05$), ** ($p < 0.01$) and *** ($p < 0.001$) *vs.* WT STD mice; $$ ($p < 0.01$) and $$$ ($p < 0.001$) *vs. Apoe*$^{-/-}$ STD mice.

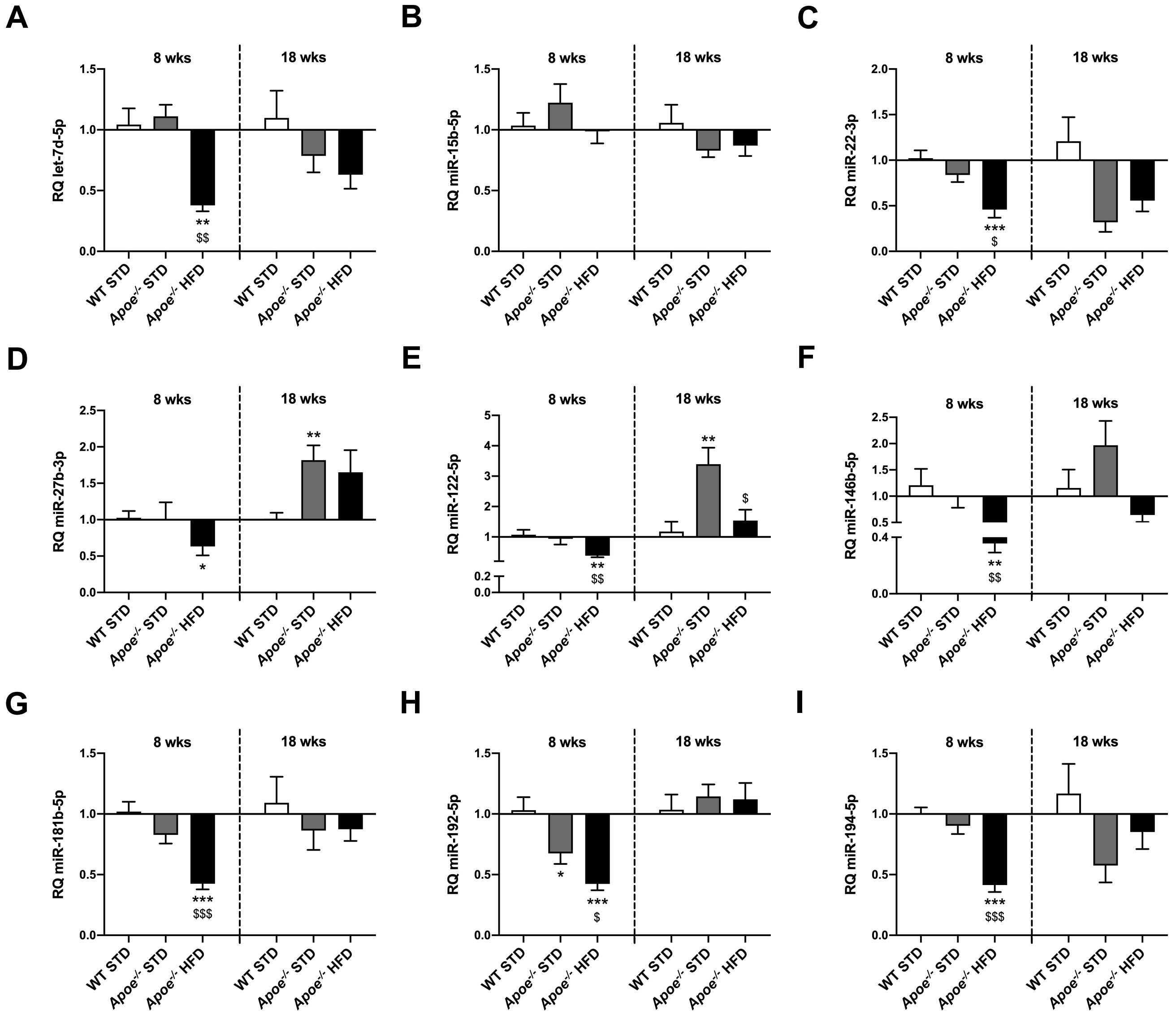

Fig. S4. miRNA expression study through RT-qPCR in the liver. Level comparison of: (A) let-7d-5p, (B) miR-15b-5p, (C) miR-22-3p, (D) miR-27b-3p (E) miR-122-5p, (F) miR-146b-5p, (G) miR-181b-5p, (H) miR-192-5p and (I) miR-194-5p in livers from WT STD (n = 5-7), *Apoe*$^{-/-}$ STD (n = 4-9) and *Apoe*$^{-/-}$ HFD (n = 6-10) mice fed 8 or 18 weeks on diet. miR-191-5p was used as a control. Results are expressed as mean ± SEM. Normality test determined 8-week of diet data as normal, except for RQ let-7d-5p, and 18-week of diet data as no normal with the exception of RQ let-7d-5p, miR-15b-5p, miR-22-3p, miR-181b-5p and miR-194-5p. For that reason, statistical significance was assessed by two-tailed unpaired Student's *t*-test in the case of normal data and no normal data were evaluated by unpaired non-parametric Mann-Whitney U test, and it was represented as * ($p < 0.05$), ** ($p < 0.01$) and *** ($p < 0.001$) *vs.* WT STD mice; $ ($p < 0.05$), $$ ($p < 0.01$) and $$$ ($p < 0.001$) *vs. Apoe*$^{-/-}$ STD mice.

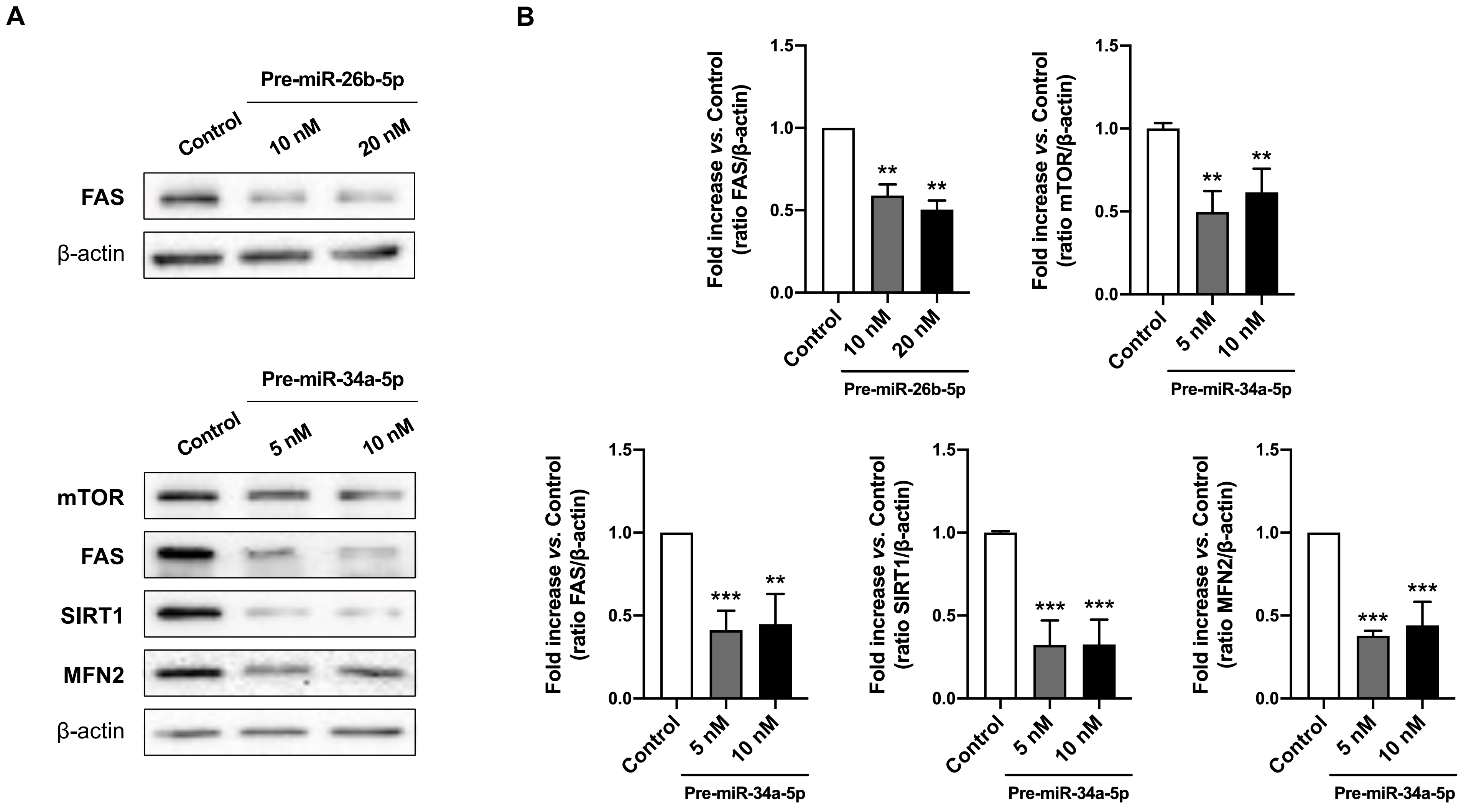

Fig. S5. ***In vitro*** **overexpression of miR-26b-5p and miR-34a-5p induces the downregulation of some of their predicted targets.** (A) Representative gels of western blot analysis of mTOR, FAS, SIRT1 and MFN2 in Huh7 cell lysates after miR-26b-5p and miR-34a-5p overexpression. β-actin was used as a loading control. (B) Histograms showing the protein/β-actin ratio quantification of band intensities in Control and transfected cells (Pre-miR-26b-5p and Pre-miR-34a-5p). At least, three technical replicates were performed. Results are expressed as mean ± SEM. Statistical significance was assessed by two-tailed unpaired Student's t-test with the exception of mTOR and FAS data which were evaluated by unpaired non-parametric Mann-Whitney U test and it was represented as ** ($p < 0.01$) and *** ($p < 0.001$) *vs.* Control cells.

Disease Models & Mechanisms • Supplementary information

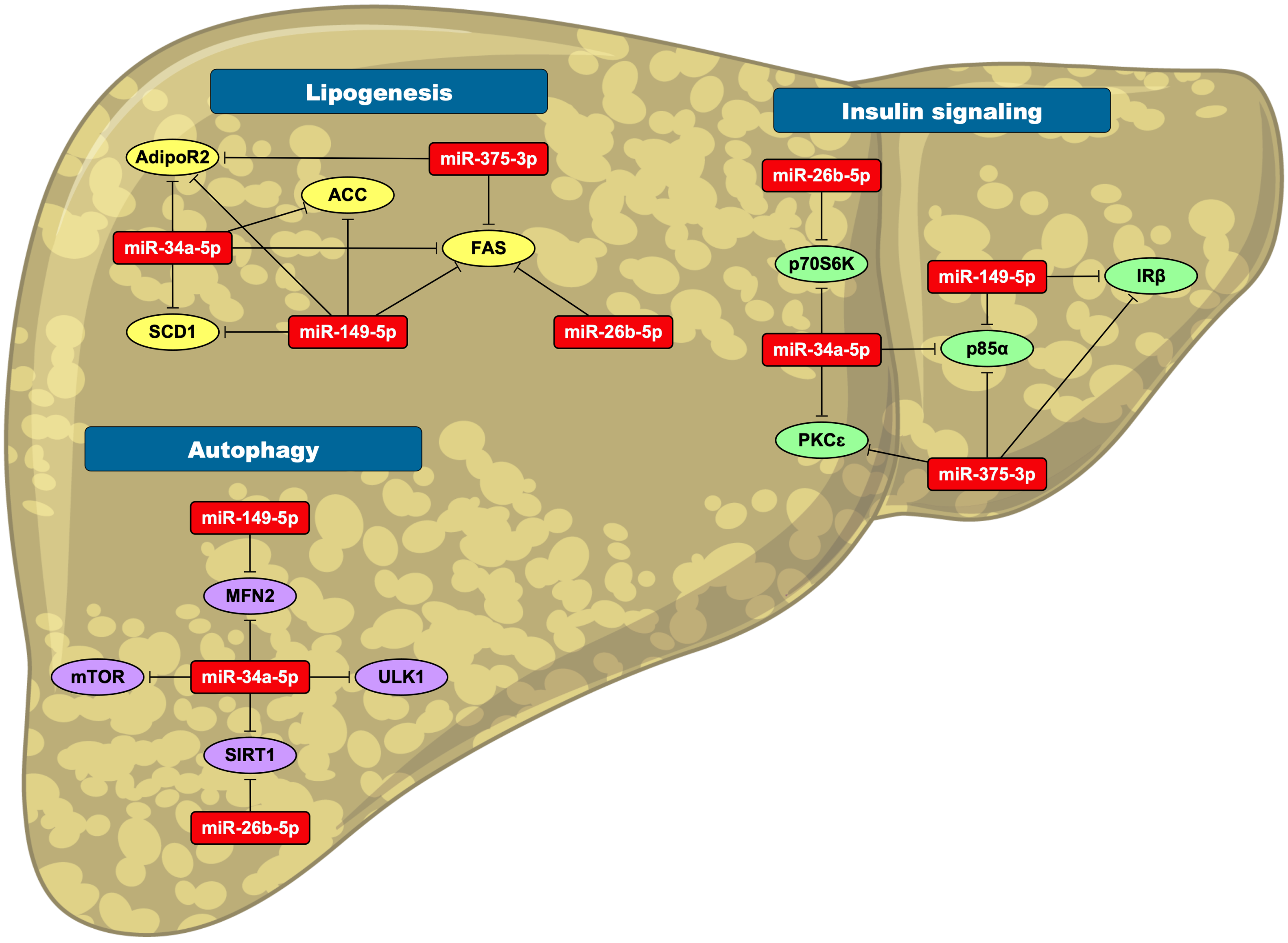

Fig. S6. Summary of miRNA-target interactions involved in NAFLD progression. The diagram displays the potential interactions of miR-26b-5p, miR-34a-5p, miR-149-5p and miR-375-5p with their targets regarding lipid metabolism, insulin signaling and autophagy.

Disease Models & Mechanisms • Supplementary information

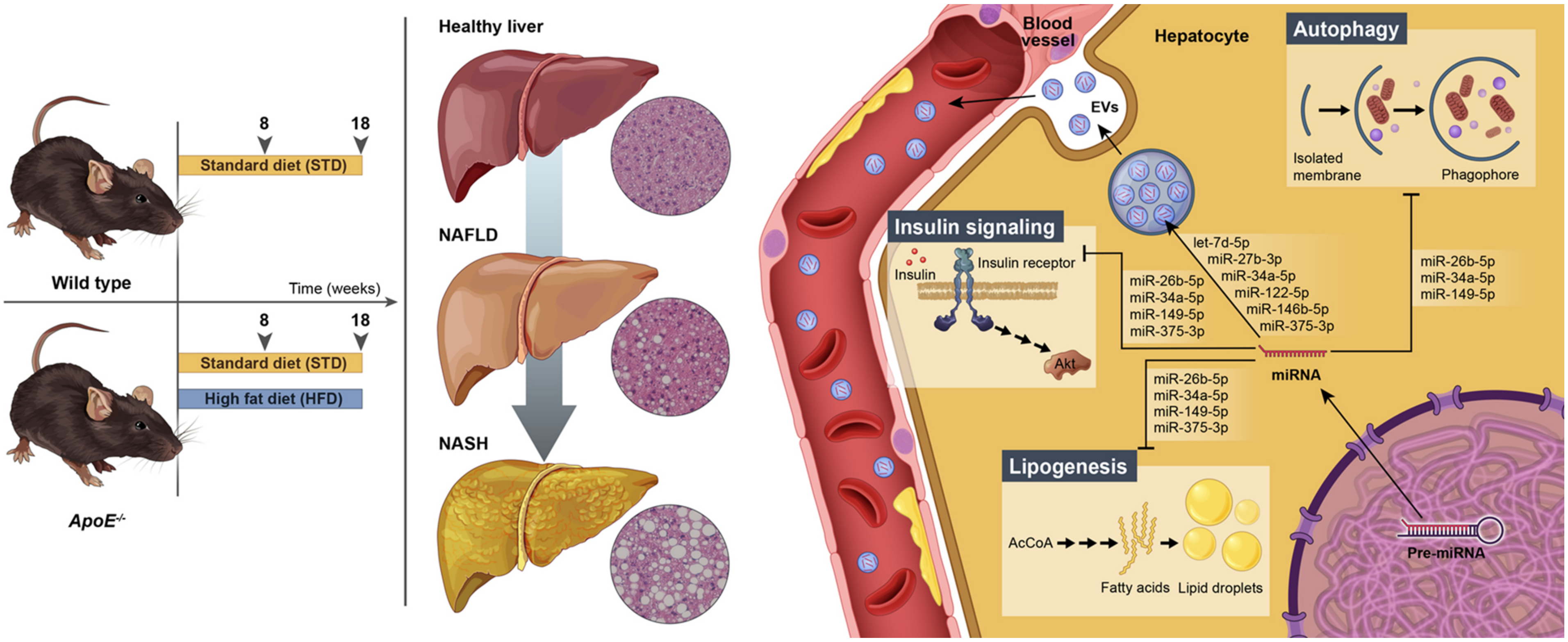

Fig. S7. Schematic representation of the main features of NAFLD in the *Apoe*$^{-/-}$ mouse model. This graph highlights the noticeable histological differences between experimental groups and the selected miRNAs as regulators of disease development and potential biomarkers for its diagnosis.

Table S1. RT-qPCR primers sequence for gene expression. Fwd.: Forward; Rev.: Reverse.

Target gene	Primer sequence
***Acaca* (NM_133360.2)**	Fwd.: 5’-GAATCTCCTGGTGACAATGCTTATT-3’<br>Rev.: 5’-GGTCTTGCTGAGTTGGGTTAGCT-3’
***Actb* (NM_007393)**	Fwd.: 5’-GACATGGAGAAGATCTGGCA-3’<br>Rev.: 5’-GGTCTCAAACATGATCTGGGT-3’
***Adipor2* (NM_197985.4)**	Fwd.: 5’-TCTCAGTGGGACATGTTTGC-3’<br>Rev.: 5’-AGGCCTAAGCCCACGAAC-3’
***Cd36* (NM_001159555.1)**	Fwd.: 5’-AGATGACGTGGCAAAGAACAG-3’<br>Rev.: 5’-CCTTGGCTAGATAACGAACTCTG-3’
***Fasn* (NM_007988.3)**	Fwd.: 5’-TCGGCGGGTCTATGCCACGA-3’<br>Rev.: 5’-GTCACCCACCTTGGTGCCCG-3’
***Ppargc1a* (NM_008904.2)**	Fwd.: 5’-TCGCTGATGCACTGCCTATG-3’<br>Rev.: 5’-GAGAGGTCCACAGAGCTGATT-3’
***Scd1* (NM_009127.4)**	Fwd.: 5’-TTCTTGCGATACACTCTGGTGC-3’<br>Rev.: 5’-CGGGATTGAATGTTCTTGTCGT-3’
***Srebf1* (NM_001358314.1)**	Fwd.: 5’-CACTGGTCGTAGATGCGGAGAA-3’<br>Rev.: 5’-TCATTGATGGAGGAGCGGTAGC-3’

Disease Models & Mechanisms • Supplementary information

Table S2. TaqMan probes used for RT-qPCR analyses.

Target gene	Reference
Ccl2	Mm00441242_m1
Gapdh	Mm03302249_g1
**mmu-let-7d-5p**	mmu478439_mir
**mmu-miR-15b-5p**	mmu482957_mir
**mmu-miR-16b-5p**	mmu482960_mir
**mmu-miR-22-3p**	mmu481004_mir
**mmu-miR-26b-5p**	mmu482965_mir
**mmu-miR-27b-3p**	mmu478270_mir
**mmu-miR-34a-5p**	mmu481304_mir
**mmu-miR-122-5p**	mmu480899_mir
**mmu-miR-146b-5p**	mmu478513_mir
**mmu-miR-149-5p**	mmu480946_mir
**mmu-miR-181b-5p**	mmu478583_mir
**mmu-miR-191-5p**	mmu481584_mir
**mmu-miR-192-5p**	mmu480876_mir
**mmu-miR-194-5p**	mmu480976_mir
**mmu-miR-375-5p**	mmu481141_mir

Table S3. List of primary antibodies used for Western blot analysis.

Antibody	Host	Dilution	Reference	Supplier
CD63 (EPR5702)	Rabbit	1:1000	ab134045	Abcam (Cambridge, UK)
MFN2	Mouse	1:1000 (liver extracts) // 1:5000 (cell lysates)	ab56889	
ACC	Rabbit	1:1000	#3662	Cell Signaling Technology (Danvers, MA, USA)
AKT	Rabbit	1:1000	#9272	
CD81 (D502Q)	Rabbit	1:1000	#10037	
FAS	Rabbit	1:1000 (liver extracts) // 1:5000 (cell lysates)	#3180	
GM130 (D6B1)	Rabbit	1:1000	#12480	
mTOR (7C10)	Rabbit	1:1000	#2983	
LC3A/B	Rabbit	1:1000	#4108	
p-AKT (Ser473) (193H12)	Rabbit	1:1000	#4058	
p-AKT (Thr308) (D25E6)	Rabbit	1:1000	#9275	
p-ULK1 (Ser757) (D70U6)	Rabbit	1:1000	#1402	
PKCε (22B10)	Rabbit	1:1000	#2683	
SCD1 (C12H5)	Rabbit	1:1000	#2794	
PI3 Kinase p85α	Rabbit	1:1000	ABS234	Merck Millipore (Burlington, MA, USA)
SIRT1	Rabbit	1:1000 (liver extracts) // 1:2000 (cell lysates)	#07-131	
IRβ (C-19)	Rabbit	1:1000	sc-711	Santa Cruz Biotechnology (Dallas, TX, USA)
α-tubulin (DM1A)	Mouse	1:1000	T6199	Sigma-Aldrich (St. Louis, MO, USA)
β-actin	Mouse	1:5000 (liver extracts) // 1:10000 (cell lysates)	A5441	

Disease Models & Mechanisms • Supplementary information
